# Anti-HIV-1 potential of *Tinospora cordifolia* extracts: Multi-targeted viral inhibition and immune landscape modulation

**DOI:** 10.1016/j.jtcme.2026.06.003

**Published:** 2026-06-11

**Authors:** R. Harshithkumar, Pratiksha Jadaun, Chandrabhan Seniya, Shraddha Y. Gaikwad, Ashwini More, Madhuri Chandane-Tak, Swapnil Borse, Preeti Chavan-Gautam, Girish Tillu, Anupam Mukherjee

**Affiliations:** a ICMR-National Institute of Translational Virology and AIDS Research, Pune, Maharashtra, India; b Department of Biotechnology, School of Basic and Applied Sciences, JSPM University, Wagholi, Pune, India; c Department of Biotechnology and Chemical Engineering, Manipal University Jaipur, Rajasthan, 303007, India; d AYUSH-Center of Excellence, CCIH-Interdisciplinary School of Health Sciences, Savitribai Phule Pune University, Pune, 411007, MH, India; e ICMR-National Institute of Virology, Pune, Maharashtra, India; f AcSIR - Academy of Scientific & Innovative Research, Ghaziabad, 201002, UP, India

**Keywords:** *Tinospora cordifolia*, Ayurveda, HIV-1, Antiviral activity, Inflammatory cytokines, Alternative therapy

## Abstract

**Background and aim:**

Despite the success of antiretroviral therapy, Human Immunodeficiency Virus type 1 (HIV-1) remains a formidable global health challenge due to drug toxicity, resistance, and persistent immune dysfunction. *Tinospora cordifolia* (TC), an Ayurvedic Rasayana herb, holds immense promise as a phytotherapeutic alternative owing to its dual antiviral and immunomodulatory potential. This study investigated the anti-HIV-1 efficacy and the mechanistic basis of TC extracts.

**Experimental procedure:**

Hydroalcoholic (HATC) and aqueous (AQTC) extracts of TC were evaluated *in vitro* using TZM-bl cells and primary human PBMCs infected with HIV-1 primary isolates. Antiviral activity, cytotoxicity, viral enzyme inhibition, and immunomodulatory effects were assessed through luciferase assays, ELISA, qRT-PCR, immunoblotting, and cytokine profiling. Molecular docking was performed to identify bioactive phytoconstituents targeting key HIV-1 enzymes.

**Results and conclusion:**

Both TC extracts exhibited potent, dose-dependent anti-HIV-1 activity, with HATC showing superior efficacy. HATC inhibited HIV-1 protease and reverse transcriptase, suppressed GAG (Group-specific Antigen), TAT (Trans-Activator of Transcription), and p24 expression, and modulated macrophage cytokine profiles by controlling IL-1β, IL-6, TNF-α, IL-10, and TGF-β. Notably, HATC may upregulate Stimulator of Interferon Genes (STING)–Interferon beta (IFN-β) signalling, suggesting enhanced innate antiviral defence. Molecular docking revealed strong binding affinities of columbin, tinosporide, and tinosporaside with HIV-1 protease and reverse transcriptase. Experimental validation confirmed that columbin demonstrated potent anti-HIV-1 activity. This is the first comprehensive study to demonstrate that TC extract exerts multi-targeted inhibition of HIV-1 replication and restores immune balance. These findings support its development as a novel adjunct phytotherapy for HIV-1 and warrant further pre-clinical investigation.

## Highlights

• *Tinospora cordifolia* extracts potently inhibit HIV-1 replication *in vitro*.
• Molecular docking reveals strong binding of phytochemicals to viral enzymes.
• Enzyme inhibition assays confirm protease and reverse transcriptase blockade.
• HATC rebalances cytokine profiles and may enhance STING-IFN-β signaling.
• Columbin demonstrates validated anti-HIV-1 activity in cellular models.

## Graphical abstract

**Figure:**
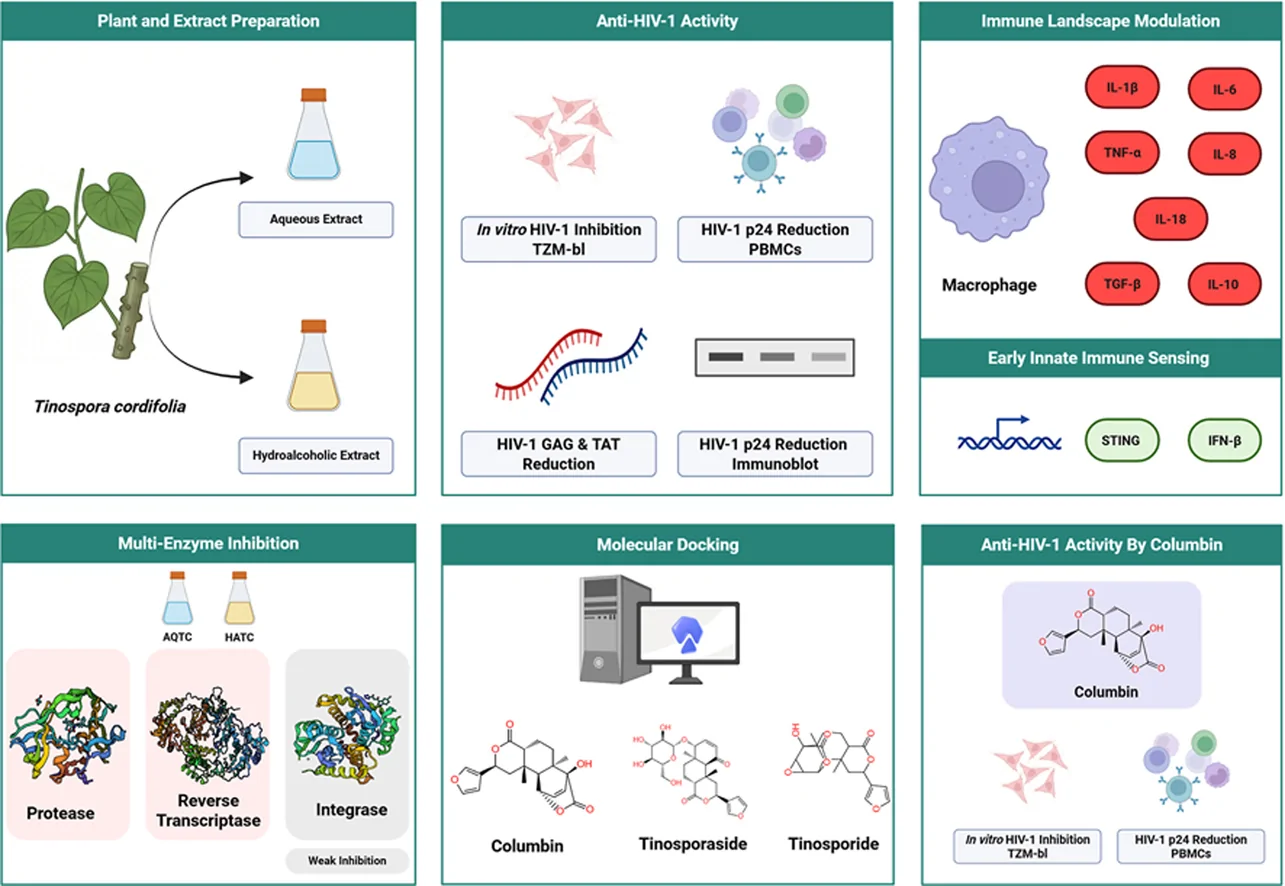

## 1 Introduction

Human Immunodeficiency Virus (HIV), an RNA virus that has developed impressive abilities in outsmarting human antiviral defences, has become a global concern and a constant in the history of pandemics. According to the data for 2024, an alarming number of 1.3 million people were added to the total number of people living with HIV, making 39.9 million people living globally.^1^ Although highly active antiretroviral therapy (HAART) has dramatically improved prospects for those living with HIV/Acquired Immunodeficiency Syndrome (AIDS), significant issues persist.^2^^,^^3^ On one side, the toxicities associated with long-term antiretroviral use pose a major challenge in the management of HIV-positive individuals, and on the other, rapidly mutating HIV evolves to become multi-drug resistant.^4^^,^^5^ The adverse effects of antiretroviral therapy span a wide spectrum, from acute toxicities like rashes to chronic complications such as hepatotoxicity, mitochondrial dysfunction, metabolic abnormalities, and lipodystrophy^6^, ^7^, ^8^, ^9^. Major clinical complications associated with HAART, including lactic acidosis, hepatic steatosis, peripheral neuropathy, myopathy, lipoatrophy, dyslipidaemia, and insulin resistance, have compelled the researchers to explore safer alternatives for the treatment of HIV infection.^10^

Natural compounds have long been attributed with safer cytotoxic profiles, as evidenced by numerous clinical trials conducted globally.^11^^,^^12^ Since these molecules have evolved naturally to perform biological functions, they tend to exhibit fewer side effects and are generally metabolized and eliminated more effectively within the body's systems compared to synthetic drugs.^13^ Natural products and their derivatives account for approximately half of the drugs approved by the FDA, as they offer an enormous diversity of molecular scaffolds and rigid structures, thereby possessing the potential to modulate and interfere with various biological functions for therapeutic purposes^14^, ^15^, ^16^, ^17^. Traditional medicine, the ancient healthcare system that served for centuries, predominantly relied on natural compounds. In recent decades, the inclusion of traditional medicine has been constantly growing as it harnesses the inherent advantages of these natural compounds, being safer yet effective.^18^^,^^19^ The World Health Organization also recognized the significance of natural compounds in medicine practices and developed a traditional medicine strategy 2014–2023, with the objective of reinforcing the role of traditional medicine in promoting and maintaining population health.^20^ The Indian system of traditional medicine, Ayurveda, has extensively explored the therapeutic potential of natural compounds.^21^ The contribution of Ayurveda in managing viral infections through its herbal remedies is extensive, and several of these herbs have been studied for their immune-enhancing and antiviral effects^22^, ^23^, ^24^. Rasayana, a rejuvenation remedy in Ayurveda, is one of the key therapies suggested for viral infections.^25^^,^^26^ According to Ayurvedic principles, rasayana botanicals possess both adaptogenic and immunomodulatory properties that can help manage viral infections.^27^^,^^28^ Thus, utilizing rasayana drugs in the management of HIV could offer dual benefits by suppressing viral replication and enhancing the immune system of the body.

*Tinospora cordifolia* (Willd.) Hook.f. and Thoms. (TC), commonly known as Guduchi or Giloy, is one of the most extensively regarded plants in Rasayana, which is quoted among the *Agrya dravyas* (principal drugs) by Charaka.^29^ This herb is a deciduous perennial climbing shrub belonging to the family Menispermaceae. The plant contains many compounds, including alkaloids (berberine, magnoflorine, palmatine), glycosides (tinocordiside, cordifolioside, tinosporaside), diterpenoid furanolactones (columbin, tinosporide), terpenoids (tinosponone, 20-hydroxyecdysone), and immunomodulatory polysaccharides that are reported for their bioactivity^30^, ^31^, ^32^, ^33^. It has wide applications as an anti-oxidant, anti-inflammatory, anti-hyperglycemic, antihyperlipidemic, hepatoprotective, neuroprotective, osteoprotective, anti-anxiety, analgesic, and antipyretic agent, as reported in various studies^30^^,^^34^, ^35^, ^36^, ^37^. Although TC is used in the treatment of various diseases, including jaundice, fever, and chronic diarrhea, its investigation in the context of HIV infection remains limited^38^, ^39^, ^40^. A randomized double-blind placebo-controlled clinical trial in 68 HIV-positive patients reported that TC extract improved clinical symptomatology and reduced eosinophil counts, suggesting its potential in HIV/AIDS management.^41^ Further, *in vitro* evaluation of TC leaf extracts in PBMCs demonstrated HIV-1 reverse transcriptase inhibitory activity, indicating the antiviral potential of this plant.^42^ These findings collectively underscore the therapeutic relevance of TC in HIV infection. However, the mechanistic basis of its antiviral activity in HIV-1 infection remains to be comprehensively characterized.

In this study, we report the *in vitro* anti-HIV activity of stem extracts of *Tinospora cordifolia* and its constituent columbin, and investigate the possible modes of inhibition through which this Rasayana herb might exert its antiviral effects. Considering the immunomodulatory and adaptogenic properties attributed to *Tinospora cordifolia* in Ayurvedic principles, exploring its therapeutic potential against HIV could offer a promising approach to suppress viral replication while enhancing the immune response.

## 2 Methods

### 2.1 Plant collection, extract preparation and bioactive compound procurement

Specimens of the plant *Tinospora cordifolia* (Willd.) were collected following standard procedures from authentic sources. Formal identification was provided by the Botanical Survey of India, Koregaon Road, Pune, where voucher specimens were deposited under the authenticated certificate number BSI/VWRC/lden.Cer./2024/0901240015667. The extraction process for TC followed established protocols with minor modifications.^43^ Briefly, TC stems were shade-dried for 7 days and then processed into fine powder through mechanical grinding. For the hydroalcoholic extract (HATC), the stem powder was immersed in a 70:30 water-alcohol solution at a ratio of 1:4 (1 part stem powder to 4 parts solvent) overnight, then extracted at 60 ± 5 °C for 3 h. The mixture was filtered three times through a 400-μm strainer, concentrated, and spray-dried to produce powder. The aqueous extract (AQTC) was prepared by macerating the stem powder in UV-filtered water overnight (8 h), followed by 8 h of gentle boiling until the volume reduced to 1/8th of the original. This concentrated liquid was filtered through cotton cloth and lyophilized to obtain a dry powder. Stock solutions were prepared by dissolving the aqueous extract in water and the hydroalcoholic extract in DMSO, with subsequent dilutions made in complete medium or phosphate-buffered saline (PBS). DMSO and alcohol concentrations were kept at non-toxic levels for cellular assays. Additionally, columbin (CAS No. 546-97-4; Merck, USA, Cat. No. SMB00086), a key diterpenoid furanolactone compound from TC was procured commercially. Columbin stock solutions were prepared in DMSO at 5 mg/mL and stored at −20 °C, with subsequent working dilutions prepared in complete cell culture medium maintaining DMSO at non-toxic levels.

### 2.2 Cellular models and viral strains

The TZM-bl cell line, a HeLa-derived recombinant cell possessing the luciferase reporter gene under the control of the HIV-1 LTR (Long Terminal Repeat) promoter, was procured from the National Institutes of Health AIDS Research and Reference Reagent Program. These cells were maintained in DMEM (Gibco, USA) supplemented with 10% FBS (Gibco, USA), HEPES buffer (Gibco, USA), and antibiotics (Sigma-Aldrich, USA) in a humidified 5% CO_2_ incubator at 37 °C. The TZM-bl cells express high levels of the CD4 receptor and the CXCR4 (X4) and CCR5 (R5) co-receptors, making them permissive to HIV-1 infection. For all screening experiments examining anti-HIV-1 activity of phytoextracts, cultures with at least 80% confluence were used.

Peripheral blood mononuclear cells (PBMCs) were obtained from a certified blood bank, where samples are screened for transfusion-transmitted infections including HIV, hepatitis B and C, and syphilis. PBMCs were isolated from blood by density gradient centrifugation using Histopaque (Sigma-Aldrich, USA). Following isolation, PBMCs were activated with phytohemagglutinin-P (PHA-P, 5 μg/mL) (Sigma-Aldrich, USA) in RPMI 1640 medium (Gibco, USA). The medium was supplemented with 10% FBS, antibiotics (50 U/mL Penicillin and 50 μg/mL Streptomycin), and 5 U/mL IL-2.

The human monocytic THP-1 cell line (ATCC: TIB-202™, ATCC, Manassas, VA, USA) was cultured in RPMI 1640 medium supplemented with 10% FBS (Gibco, USA), 1% penicillin-streptomycin (Sigma-Aldrich, USA), 20 mM HEPES (Gibco, USA), and 1 mM sodium pyruvate (Gibco, USA). Cells were maintained in a humidified incubator at 37 °C with 5% CO_2_ and passaged at approximately 80% confluency. For macrophage differentiation, THP-1 cells were seeded at a density of 1 × 10^6^ cells per well and treated with 20 nM phorbol 12-myristate 13-acetate (PMA) for 24 h. After treatment, PMA-containing medium was removed, cells were washed twice with PBS, and fresh RPMI 1640 medium with 10% FBS was added. Cells were then allowed to rest for an additional 24 h before further experimentation.

The Indian primary isolate HIV-1_VB028_ (R5, Subtype C), was cultured at the Division of Virology, ICMR-National Institute of Translational Virology and AIDS Research, Pune, while the HIV-1_UG070_ (X4, Subtype D) primary isolate was obtained from the NIH ARRRP. Both HIV-1 strains were propagated in PHA-P activated PBMCs, and viral infectivity was quantified using the HIV-1 p24 antigen detection assay (Abcam, UK). The TZM-bl cell line served as the platform for viral titration, and the 50% tissue culture infective dose (TCID_50_) was calculated using the Spearman-Karber method as previously described.^44^^,^^45^

### 2.3 Evaluation of cytotoxicity

The *in vitro* cytotoxic profiles of TC extracts (AQTC and HATC) were determined using the MTT assay in TZM-bl cells and activated PBMCs. TZM-bl cell monolayers (1 × 10^4^ cells/well) were cultured overnight in 96-well plates before exposure to serial dilutions of the AQTC (16-0.125 mg/mL) and HATC (4-0.125 mg/mL) extracts. Triton X-100 (1% v/v) treated cells were included as a positive cytotoxicity control, while untreated cells served as negative controls. After 48 h of incubation, 20 μL of MTT reagent (5 mg/mL) was added to determine cell viability colorimetrically. For PBMC cytotoxicity assessment, activated cells were treated with varying concentrations of extracts and incubated for 5 days at 37 °C in a humidified 5% CO_2_ incubator, followed by MTT-based viability assessment. Optical density (OD) was measured at 550 nm with a reference wavelength of 630 nm using a multimode reader, and corrected absorbance values were obtained as:

$${OD}_{corrected}={OD}_{550}-{OD}_{630}$$

Percentage cell viability was then calculated using the formula:

$$\%\;Viability=\frac{{OD}_{sample}-{OD}_{blank}}{{OD}_{control}-{OD}_{blank}}\times 100$$

To determine the 50% cytotoxic concentration (CC_50_), defined as the extract concentration causing a 50% reduction in cell viability, logarithmic regression analysis was performed. Specifically, percentage cell viability values were plotted against their corresponding extract concentrations, and a logarithmic trendline was applied to the data to generate the regression equation:

y=m·ln(x)+C

where y represents percentage cell viability, x represents extract concentration (mg/mL), m is the slope coefficient, and C is the y-intercept.

To obtain the CC_50_ value, y was set to 50 in the regression equation, and the corresponding concentration (x) was calculated as:

$${CC}_{50}=\exp\left(\frac{50-C}{m}\right)$$

### 2.4 *In vitro* anti-HIV-1 assays

The antiviral activity of HATC and AQTC extracts against HIV-1 primary isolates was evaluated at sub-cytotoxic concentrations determined by the MTT assay.^46^ For cell-associated virus inhibition, TZM-bl cells (1 × 10^4^ cells/well) were first infected with pre-titrated HIV-1_VB028_ and HIV-1_UG070_ stocks for 2 h, then exposed to serial dilutions of HATC and AQTC (1.0-0.0078 mg/mL). Appropriate vehicle control containing equivalent volumes of DMSO (without extract) was included in all experiments. Luciferase activity was measured after 48 h using Britelite plus reagent (PerkinElmer, USA). Dose-dependent percentage inhibition was calculated using the formula below:

$$Percentage\;Inhibition=\left[1-\frac{\left({Avg\;RLU}_{Test}-{Avg\;RLU}_{Mock}\right)}{\left({Avg\;RLU}_{VC}-{Avg\;RLU}_{Mock}\right)}\right]\times 100$$

where Avg RLU_Test_ represents the average relative light units from extract-treated infected cells, Avg RLU_VC_ represents the infected cells without extract treatment, and Avg RLU_Mock_ represents uninfected cells.

The 50% effective concentration (EC_50_) values were subsequently calculated and compared with Azidothymidine (AZT).^47^ For cell-free virus inhibition, viral stocks were pre-treated with serial dilutions of the extracts (2.0-0.0156 mg/mL) for 1 h before addition to TZM-bl cells. Equivalent volumes of DMSO served as the vehicle control. Relative luciferase activity was measured 48 h post-infection (hpi), and percentage inhibition and EC_50_ values were determined as described above and compared with dextran sulphate (DS). All experiments were performed independently in triplicate.

### 2.5 Inhibition confirmatory assays

To confirm anti-HIV-1 activity in primary target cells, activated PBMCs (0.2 × 10^4^ cells/well) were infected with pre-titrated HIV-1_VB028_ (40 TCID_50_) and treated with sub-toxic concentrations of the extracts (1.0-0.0078 mg/mL). Vehicle controls with equivalent DMSO volumes were included to rule out solvent effects. After 5 days, HIV-1 p24 antigen levels in the supernatant were quantified by ELISA. Absorbance was measured at 450 nm, and EC_50_ values were subsequently calculated, with AZT as the reference anti-HIV drug. For the virucidal cell-free confirmatory assay, HIV-1_VB028_ (40 TCID_50_) was treated with serial dilutions of the extracts (2.0-0.0156 mg/mL) for 1 h before addition to activated PBMCs. After 24 h, cells were washed, resuspended in fresh medium, and incubated for 5 days. Supernatants were tested for p24 antigen, and EC_50_ values were calculated with DS serving as a positive control.

### 2.6 Protein structure selection and preparation

HIV-1 enzymes, including HIV-1 protease (PDB 5KR0, resolution 1.8 Å), HIV-1 reverse transcriptase (PDB 3QO9, resolution 2.09 Å), and HIV-1 integrase (PDB 1QS4, resolution 2.01 Å), were retrieved as crystal structures from the RCSB Protein Data Bank (http://www.rcsb.org). Protein preparation was conducted using BIOVIA Discovery Studio Visualizer v21.1.0.20298 or AutoDockTools-1.5.7 (https://www.3ds.com/products-services/biovia/products/molecular-modeling-simulation/biovia-discovery-studio/), which involved removing water molecules, heteroatoms, and co-crystallized ligands to obtain clean protein structures. Further optimization included adding and optimizing polar hydrogens, merging non-polar hydrogens, energy minimization with Kollman charges, repairing missing atoms, and other refinements as previously described.^48^^,^^49^

### 2.7 Ligand selection and preparation

Bioactive compounds from TC were selected for docking analysis based on previously published phytochemical identification.^31^ The selected compounds included 20-hydroxy-ecdysone (CID:5459840), berberine (CID:2353), columbamine (CID:72310), columbin (CID:188289), magnoflorine (CID:73337), menisperine (CID:161487), syringin (CID:5316860), tinocordiside (CID:177384), tinosporaside (CID:14194109), and tinosporide (CID:167631). Both 2D and 3D structures of these compounds were retrieved from the PubChem database (https://pubchem.ncbi.nlm.nih.gov) in SDF format. Using Avogadro tool,^50^ the 2D structures were converted to 3D conformations by applying appropriate tautomeric, stereochemical, and ionization modifications along with energy minimization and flexible filtering to generate the most stable conformations for docking studies.

### 2.8 Molecular docking and analysis

Molecular docking simulations were conducted to assess the inhibitory potential of the selected phytochemicals against three HIV-1 enzymes. AutoDockTools 1.5.7 (https://autodock.scripps.edu) was utilized for preparing both protein and ligand files for docking. Protein preparation involved adding polar hydrogens, merging non-polar hydrogens, and assigning Kollman charges. For ligands, Gasteiger charges were applied for optimization of the energy state. Torsion trees were carefully examined to select appropriate torsion settings in AutoDock v4.2.^51^ Using AutoGrid4, a grid box of 60 × 60 × 60 Å with 0.5 Å spacing was generated, centered on the active site residues of each protein target. The Lamarckian Genetic Algorithm 4.2 was employed to run docking simulations with 10 runs over 150 populations using default parameters, allowing comprehensive exploration of potential binding conformations and interactions between ligands and protein targets.

The docking results were analyzed based on binding energy (kcal/mol), inhibition constant (Ki), and molecular interactions, including hydrogen bonds, hydrophobic interactions, π-alkyl interactions, and π-sigma interactions. A 4 Å cut-off radius was used to check interactions in the catalytic site. The PMV tool in AutoDockTools v1.5.7 was used for conformational analysis and visualization of molecular interactions, while BIOVIA Discovery Studio Visualizer v21.1.0.20298 generated two-dimensional interaction plots to better understand binding modes and key residue interactions. Compounds were ranked for each protein target according to their binding energy values, with more negative values indicating stronger binding affinity.

### 2.9 HIV-1 protease inhibition fluorometric assay

The HIV-1 protease inhibitory potential of the extracts was evaluated using a fluorometric screening assay (Abcam, UK) based on cleavage of a synthetic peptide substrate, releasing a fluorescent moiety. Extracts at their respective EC_80_ concentrations were diluted to 10 times the desired concentration, then incubated with HIV-1 protease in a 96-well plate for 15 min. Fluorescence measurements (330/450 nm excitation/emission) were taken immediately after substrate addition and monitored in kinetic mode for 4 h at 37 °C. Pepstatin (1 mM) served as an inhibitor control, and DMSO (1% v/v) was employed as a solvent control. Ritonavir (10 μM) was selected as the additional positive control due to its status as a clinically established and well-documented HIV-1 protease inhibitor, with the concentration based on published literature values. Percent relative inhibition was calculated based on relative fluorescence units compared to the enzyme control.

### 2.10 Colorimetric reverse transcriptase assay for HIV-1 reverse transcriptase inhibition

The inhibitory effects of HATC and AQTC extracts on HIV-1 reverse transcriptase were evaluated using the colorimetric reverse transcriptase assay kit (Roche, Germany). Extracts at their respective EC_80_ concentrations were pre-incubated with recombinant HIV-1 reverse transcriptase for 1 h at 37 °C, along with a reaction mixture containing digoxigenin- and biotin-labelled nucleotides and a template/primer hybrid. Vehicle control with equivalent DMSO volume was included alongside extract treatments. The reaction mixtures were then transferred to streptavidin-coated microwells and incubated for an additional hour to facilitate binding of the biotin-labelled, newly synthesized DNA to the immobilized streptavidin. Subsequently, an anti-digoxigenin antibody conjugated with horseradish peroxidase (HRP) was added to each well and incubated for 1 h to allow binding to the digoxigenin-labelled DNA. After washing, the HRP substrate was introduced, and the resulting colorimetric reaction was monitored by measuring absorbance at 405 nm with a reference wavelength of 490 nm using a microplate reader. AZT, a known HIV-1 reverse transcriptase inhibitor, served as a positive control in the assay.

### 2.11 HIV-1 integrase catalytic activity inhibition assay

The inhibitory effects of HATC and AQTC extracts on HIV-1 integrase catalytic activity were assessed using a commercially available non-radioactive assay kit (XpressBio, USA) in accordance with the manufacturer's protocol. The assay utilizes a streptavidin-coated 96-well plate with immobilized biotinylated double-stranded HIV-1 LTR U5 donor substrate oligonucleotides. Recombinant full-length HIV-1 integrase protein was pre-incubated with the TC extracts at their respective EC_80_ concentrations before introduction to wells containing donor substrate DNA. Appropriate vehicle control containing equivalent DMSO volume was included in parallel. The integration reaction was initiated by the addition of a double-stranded target substrate (TS) DNA containing a 3′-end modification. The integrase-mediated strand transfer process involves the endo-nucleolytic cleavage of two nucleotides from the exposed 3′ terminus of the HIV-1 LTR DS DNA, followed by the integration of the processed DS into the TS DNA. Detection employed an HRP-conjugated antibody specific for the TS DNA 3′-end modification, with subsequent colorimetric quantification at 450 nm. Experimental controls included sodium azide (positive control) provided with the kit, the established integrase inhibitor raltegravir (RAL, 0.48 μM) as a reference compound, and integrase enzyme (100% activity control) in the absence of inhibitors.

### 2.12 Quantitative real-time PCR (qRT-PCR)

RNA extraction was performed using TRIzol™ reagent (Invitrogen, USA) from THP-1-derived macrophages according to the manufacturer's instructions. Following spectrophotometric assessment of RNA quality and concentration, cDNA synthesis was conducted by reverse transcribing 1 μg of total RNA using the SuperScript™ III First-Strand Synthesis System (Invitrogen, USA) with random hexamer primers. Quantitative PCR analysis was carried out on a real-time PCR (CFX96) system using Power SYBR™ Green PCR Master Mix (Invitrogen, USA) with gene-specific primers (Supplementary Table 1). GAPDH was employed as the internal control for normalization. Relative gene expression was determined using the 2^−ΔΔCt^ method, with all reactions performed in triplicate.

### 2.13 Immunoblotting

Following experimental endpoints, cells were washed with ice-cold PBS and lysed using RIPA buffer containing protease inhibitors. Protein extracts were separated by electrophoresis on 10% SDS-PAGE gels and transferred to nylon membranes. The membranes were blocked with 5% non-fat dried milk for 1 h at room temperature to prevent non-specific binding. The blocked membranes were incubated overnight at 4 °C with rabbit HIV p24 antibody (1:1000 dilution; Abcam, Cambridge, UK). After three washes with TBST, membranes were incubated with HRP-conjugated anti-rabbit secondary antibody (1:2000; Cell Signaling Technology) for 1 h at room temperature. Protein bands were visualized using Pierce™ ECL Western Blotting Substrate (ThermoFisher Scientific) and detected with a ChemiDoc imaging system (BioRad). HRP-tagged GAPDH antibody (Santa Cruz Biotechnology) was used to account for variations in protein loading. The protein band intensities were measured using the ImageJ software v1.53a (NIH) and were normalized with GAPDH.

### 2.14 Statistical analysis of *in vitro* assays

All *in vitro* experiments were conducted in a minimum of three technical replicates, and the average values were used for analysis. Data were presented as percentage inhibition relative to untreated controls, following background correction. Each experiment was independently repeated at least three times, and results were expressed as mean ± standard deviation (SD). Statistical significance across varying concentrations of HATC or AQTC and among different experimental conditions was assessed using one-way analysis of variance (ANOVA) in GraphPad Prism software. Differences were considered statistically significant at ∗*p* < 0.05*, ∗∗p* < 0.01, and *∗∗∗p* < 0.001.

## 3 Results

### 3.1 *In vitro* cytotoxicity profiles of TC extracts on TZM-bl cell line and PBMCs

The cytotoxicity of AQTC and HATC was assessed on the TZM-bl cell line and PBMCs using the MTT assay. As shown in Fig. 1, both extracts exhibited relatively low cytotoxicity toward the examined cellular models, though AQTC demonstrated lesser cytotoxic effects compared to HATC across both cell types. In TZM-bl cells, the CC_50_ values were determined to be 3.80 mg/mL for AQTC and 2.01 mg/mL for HATC (Fig. 1A and B). When comparing cell types, TZM-bl cells generally tolerated higher extract concentrations than PBMCs. Specifically, PBMCs exhibited substantially higher sensitivity to AQTC extract with a CC_50_ of 1.96 mg/mL, demonstrating almost twice the sensitivity compared to TZM-bl cells (Fig. 1C). However, PBMC's sensitivity to HATC extract with CC_50_ of 1.74 mg/mL was only marginally greater than that observed in TZM-bl cells (Fig. 1D). A clear concentration-dependent decrease in cellular viability was observed in both cell types, with the corresponding mean CC_50_ values presented in Fig. 1E. Concentrations below the determined CC_50_ values were selected for the subsequent anti-HIV-1 screening assay.

**Fig. 1:**
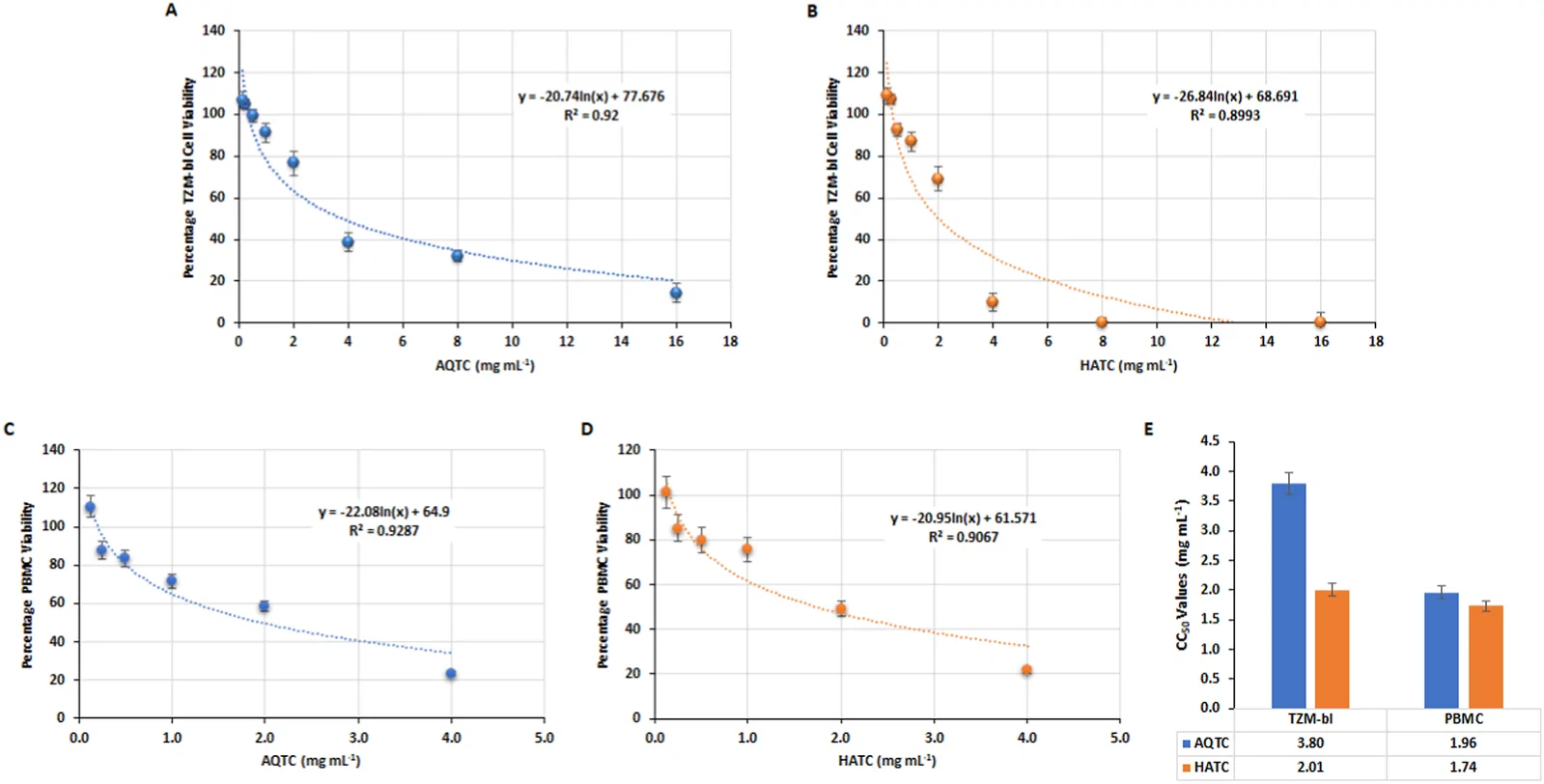
Determination of cytotoxic concentration of *Tinospora cordifolia* aqueous (AQTC) and hydroalcoholic (HATC) extracts. The percentage cell viability was examined for (**A**) AQTC and (**B**) HATC on TZM-bl cells at 0.125 - 16.0 mg/mL concentrations. The extract concentration versus percentage viability of PBMCs were also tested for (**C**) AQTC and (**D**) HATC extracts in a dose-dependent kinetics (0.125 - 4.0 mg/mL). (**E**) Mean CC_50_ values of AQTC and HATC were determined and represented as error bar with SD from three independent assays.

### 3.2 *In vitro* HIV-1 inhibition by the TC extracts

The anti-HIV-1 activity of the plant extracts HATC and AQTC was evaluated against two distinct clades of HIV-1 strains representing CCR5 (R5) and CXCR4 (X4) tropisms. Initial screening was conducted in the TZM-bl reporter cell line, followed by validation of the antiviral effects in physiologically relevant PBMCs. All testing was conducted at extract concentrations below their respective cytotoxic thresholds, as determined by CC_50_ values.

#### 3.2.1 Anti-HIV activity of the aqueous extract of TC

The anti-HIV activity of the AQTC was evaluated using cell-associated and cell-free assays. Initially, the TZM-bl cells pre-infected with HIV-1 primary isolates HIV-1_VB028_ and HIV-1_UG070_ were treated with varying concentrations of AQTC. The results demonstrated a dose-dependent inhibition of HIV-1 infection in the cell-associated assay for both clades (Fig. 2A). The EC_50_ values of AQTC in the cell-associated assay were significantly different between the two viral strains, with 0.01 mg/ml for HIV-1_VB028_ compared to 0.71 mg/ml for HIV-1_UG070_ (Supplementary Fig. 1A). Further, in the cell-free assay, where AQTC was pre-incubated with the virus, the extract exhibited excellent inhibition of viral infection in TZM-bl cells (Fig. 2B). The inhibition was more consistent between the strains, with EC_50_ values of 0.51 mg/ml and 0.48 mg/ml for HIV-1_VB028_ and HIV-1_UG070_, respectively (Supplementary Fig. 1B).

**Fig. 2:**
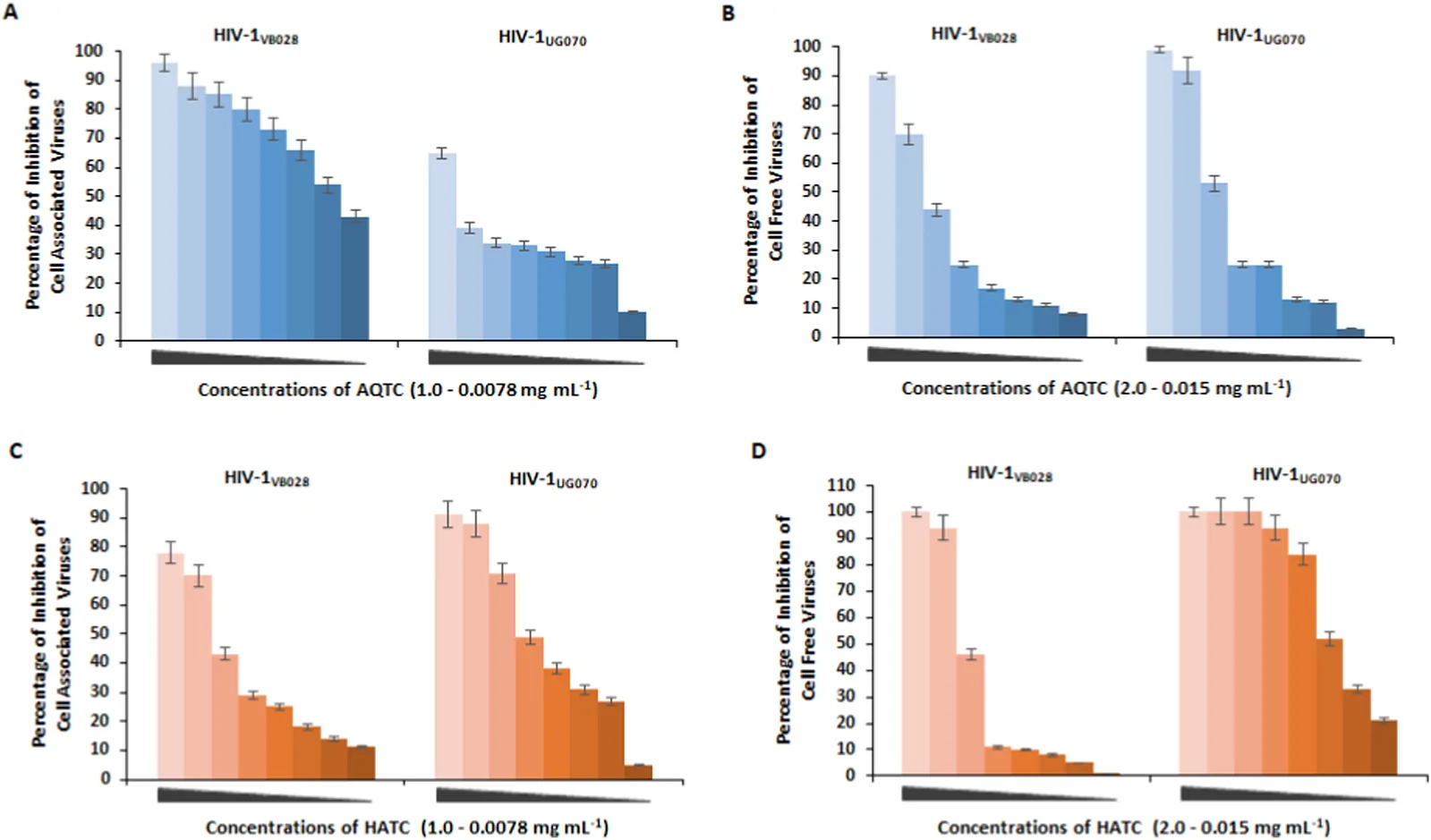
Percentage inhibition of HIV-1 replication by the *Tinospora cordifolia* extracts in TZM-bl cells. **(A)** Cell-associated and **(B)** cell-free assays treated with *Tinospora cordifolia* aqueous extract (AQTC) on HIV-1_VB028_ (R5, Subtype C) and HIV-1_UG070_ (X4, Subtype D) infected cells. Treatment of *Tinospora cordifolia* hydroalcoholic extract (HATC) through **(C)** cell-associated and **(D)** cell-free assays on HIV-1_VB028_ and HIV-1_UG070_ infected cells.

#### 3.2.2 Anti-HIV activity of the hydroalcoholic extract of TC

The HATC also demonstrated significant antiviral activity against HIV-1 when assessed in the TZM-bl cell line. In cell-associated assays, HATC potently inhibited viral infection in pre-infected TZM-bl cells for both viral clades tested (Fig. 2C). The extract exhibited EC_50_ values of 0.29 mg/ml against HIV-1_VB028_ and 0.13 mg/ml against HIV-1_UG070_, demonstrating effective inhibition of both viral strains (Supplementary Fig. 1A). Additionally, in cell-free assays with TZM-bl cells, HATC preincubated with HIV-1 similarly demonstrated strong inhibitory effects (Fig. 2D), exhibiting EC_50_ values of 0.53 mg/ml against HIV-1_VB028_ and a remarkably lower 0.05 mg/ml against HIV-1_UG070_ (Supplementary Fig. 1B).

### 3.3 Validation of anti-HIV activity of extracts in PBMCs

The antiviral efficacy of both TC extracts was further validated in PBMCs, which represent a physiologically relevant model for HIV infection. When evaluated against HIV-1_VB028_, both extracts demonstrated dose-dependent inhibition of viral replication as measured by p24 antigen levels. AQTC exhibited effective inhibition in PBMCs with an EC_50_ value of 0.083 mg/ml in cell-associated assays (Fig. 3A and Supplementary Fig. 2A). In cell-free assays, AQTC showed even greater potency with an EC_50_ value of 0.012 mg/ml (Fig. 3B and Supplementary Fig. 2B). Similarly, HATC demonstrated comparable inhibitory activity in PBMCs with an EC_50_ value of 0.082 mg/ml in cell-associated assays (Fig. 3C and Supplementary Fig. 2A). When tested in cell-free conditions, HATC exhibited exceptional neutralization against HIV-1_VB028_ with an EC_50_ value of 0.009 mg/ml (Fig. 3D and Supplementary Fig. 2B). These findings confirm that both extracts maintain their antiviral efficacy in primary human cells, supporting their potential as candidates against HIV-1.

**Fig. 3:**
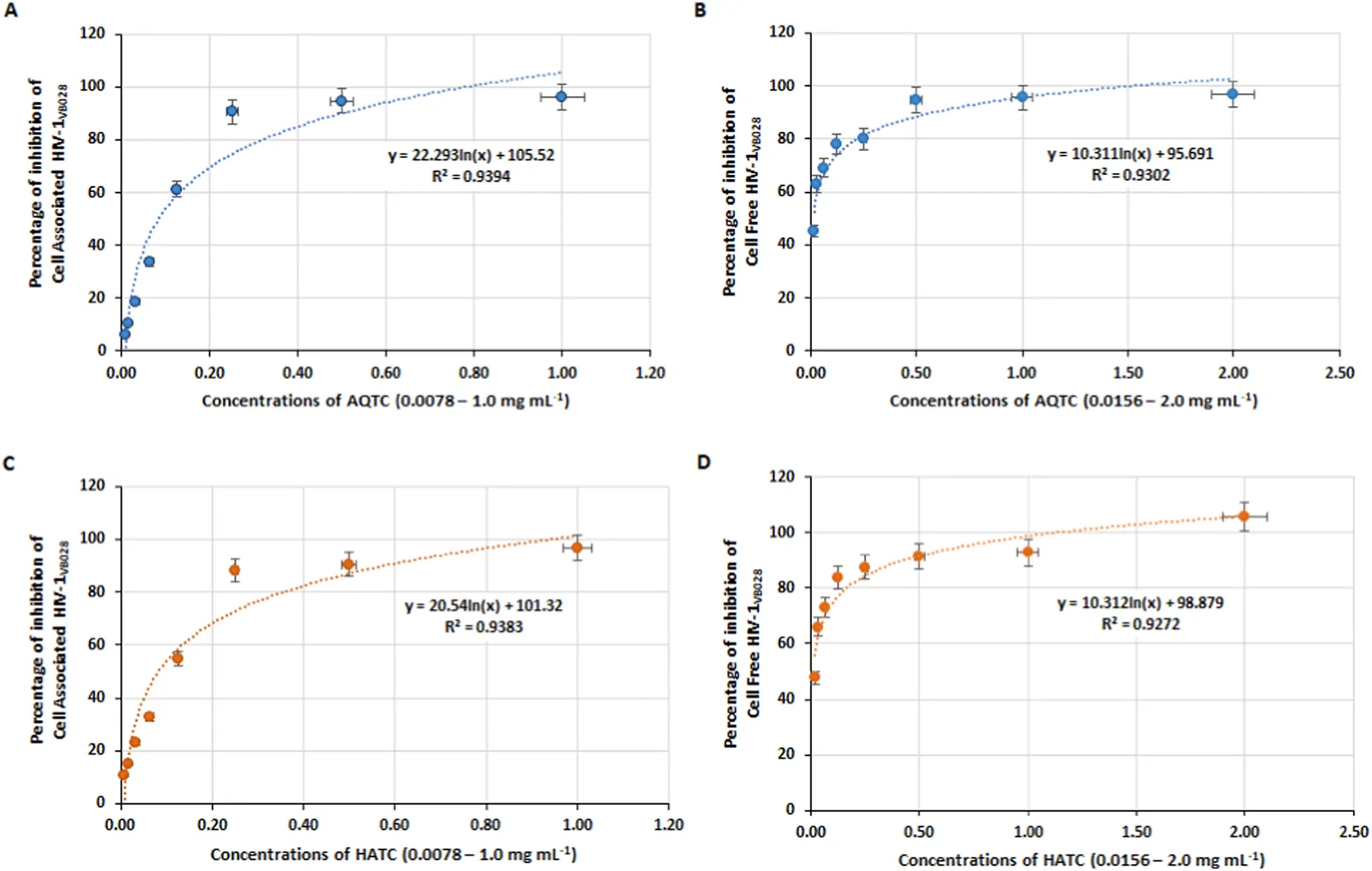
Anti-HIV-1 activity of *Tinospora cordifolia* extracts in HIV-1_VB028_ infected PBMCs. Dose-dependent inhibition of HIV-1 infection in **(A)** cell-associated and **(B)** cell-free assays by *Tinospora cordifolia* aqueous extract (AQTC). Dose-dependent inhibition of HIV-1 infection in **(C)** cell-associated and **(D)** cell-free assays by *Tinospora cordifolia* hydroalcoholic extract (HATC). The results shown are the means of at least three experimental replicates plus the standard deviations were calculated and represented as the error bar.

### 3.4 Molecular interaction analysis of TC bioactive compounds against HIV-1 targets

The molecular docking analysis was conducted to evaluate the binding interactions and inhibitory potential of ten phytochemical compounds against three critical HIV-1 target proteins: protease (5KR0), reverse transcriptase (3QO9), and integrase (1QS4) (Supplementary Table 2–4). The docking analysis assessed various binding parameters, including binding energy (kcal/mol), inhibition constant (Ki), and specific biomolecular interactions with amino acid residues via hydrogen bonds, hydrophobic interactions, π-alkyl bonds, and π-sigma/π-anion bonds. The binding energy values ranged from −4.56 to −9.04 kcal/mol across all compound-protein combinations, with corresponding inhibition constants (Ki) at different micromolar levels, indicating varying degrees of binding affinity. The inhibition constant (Ki), representing the theoretical strength of inhibition, was calculated using the equation $K_{i}=\exp\left(\frac{\Delta G}{RT}\right)$, where ΔG represents the Gibbs free energy of binding, R is the gas constant (8.314 J mol^−1^ K^−1^), and T is the absolute temperature at 298.15 K^51^. The tables represent the detailed molecular interaction profiles of all tested compounds with the HIV-1 target proteins, highlighting the specific amino acid residues involved in different types of interactions. The tabulated data reveal that most compounds demonstrated multiple types of interactions with the target proteins, including hydrogen bonding with catalytically important residues, extensive hydrophobic contacts, and π-interactions that collectively contribute to binding stability. Notably, the interactions with HIV-1 integrase were generally weaker compared to those with protease and reverse transcriptase, with binding energies ranging from −4.56 to −6.58 kcal/mol (Supplementary Table 4). None of the TC compounds demonstrated a strong molecular interaction against integrase. This relatively poor binding to integrase can be attributed to fewer specific interactions with catalytically important residues and a less optimal geometric fit within the integrase active site. Among the tested compounds, columbin (CID_188289), tinosporaside (CID_14194109), and tinosporide (CID_167631) demonstrated particularly notable binding affinities toward the key amino acids available in HIV-1 protease and reverse transcriptase, suggesting their potential to render structural rearrangements, which may lead to effective inhibition of HIV-1 infection. These compounds exhibited binding energies ranging from −8.46 to −9.04 kcal/mol against protease (Supplementary Table 2), and −8.28 to −8.98 kcal/mol against reverse transcriptase (Supplementary Table 3), indicating strong and stable molecular associations with key amino acids found in the binding pocket of these essential viral enzymes.

#### 3.4.1 Molecular interactions of columbin with HIV-1 protease and reverse transcriptase

Columbin, a key diterpenoid furanolactone compound from TC, exhibited exceptional binding affinity against HIV-1 protease with a binding energy of −9.04 kcal/mol and an inhibition constant (Ki) of 0.23 μM, making it the strongest binder among all tested compounds for this target (Fig. 4A). The molecular interaction analysis revealed that columbin forms multiple hydrogen bonds with critical amino acid residues of the protease active site, including ASP29, ASP30, ILE47, GLY48, and ILE50. Additionally, columbin established extensive hydrophobic interactions with LYS45, GLY52, PHE53, LEU76, THR80, and ILE84, while demonstrating π-alkyl interactions with ALA28, VAL32, and ILE54.

**Fig. 4:**
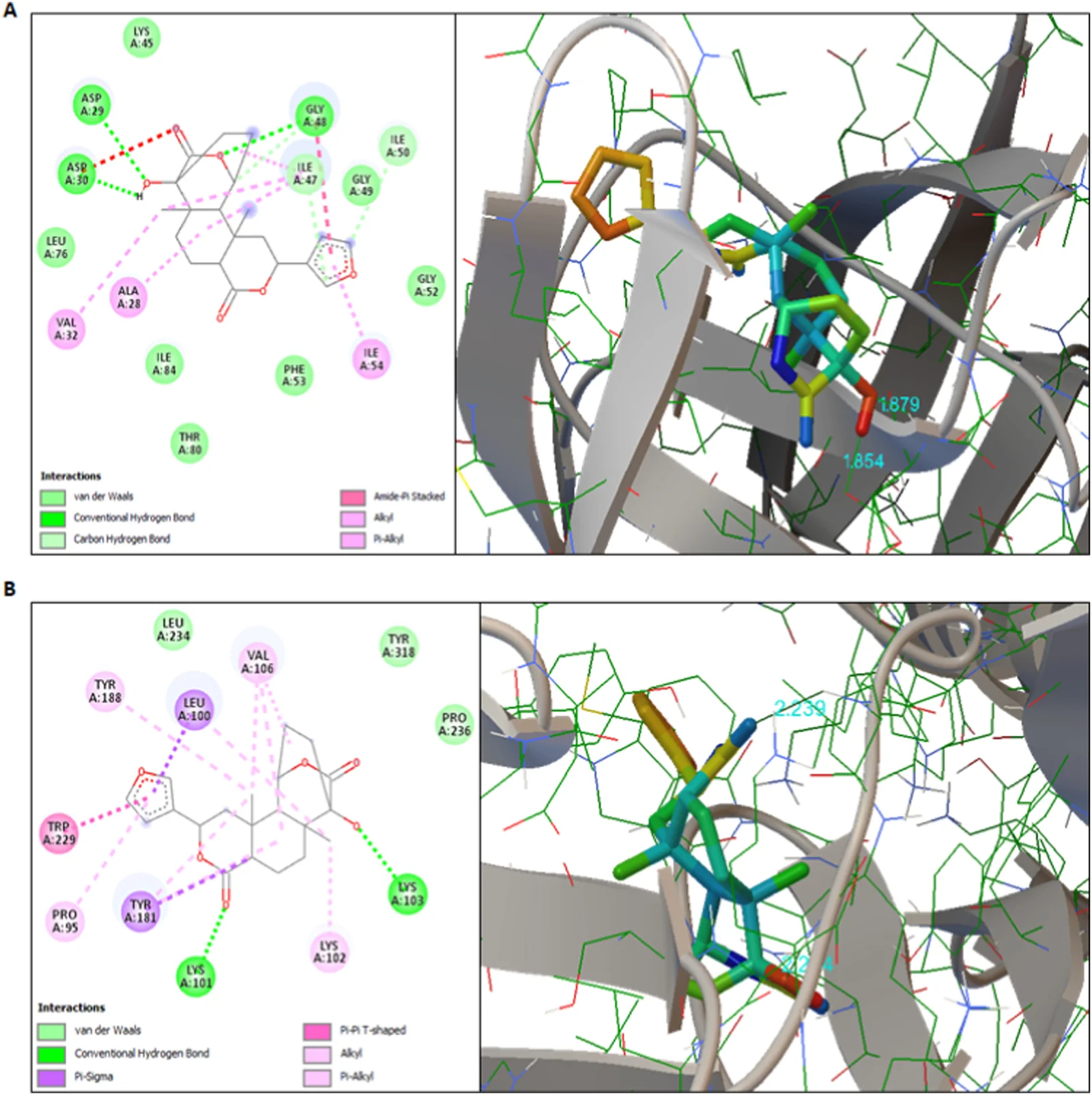
Molecular interactions between phytomolecule of *Tinospora cordifolia,* columbin (CID_188289) with HIV-1 key proteins. 2D interaction plot (left panel) and 3D map (right panel) of molecular docking simulaiton after **(A)** protease (PDB 5KR0) and **(B)** reverse transcriptase (PDB 3QO9).

When docked with HIV-1 reverse transcriptase, columbin displayed a binding energy of −8.28 kcal/mol with an inhibition constant of 0.85 μM. The compound forms hydrogen bonds with LYS101 and LYS103, which are critical residues in the reverse transcriptase enzyme active site (Fig. 4B). The stability of the complex is enhanced by hydrophobic interactions with LEU234, PRO236, and TYR318, along with π-alkyl interactions with multiple residues, including PRO95, LYS102, VAL106, TYR188, and TRP229. π-sigma interactions with LEU100 and TYR181 further strengthen the overall binding profile.

The interaction profile of columbin showed strong binding to protease coupled with significant affinity for reverse transcriptase, indicating that columbin may be an effective dual-target inhibitor of HIV-1 replication. Compared to FDA-approved drugs, columbin demonstrates superior binding energy against HIV-1 protease than Ritonavir (−4.35 kcal/mol) and stronger binding against HIV-1 reverse transcriptase than Zidovudine (−7.23 kcal/mol).^48^

To validate the computational predictions, commercially available columbin was evaluated for its cytotoxic profile and antiviral efficacy in cellular models. The cytotoxicity assessment using the MTT assay revealed a CC_50_ of 1.08 mg/mL in TZM-bl cells and 0.78 mg/mL in PBMCs, with TZM-bl cells demonstrating approximately 1.4-fold lower sensitivity compared to primary cells (Fig. 5A–C). Cell viability remained above 95% at concentrations up to 0.15625 mg/mL in TZM-bl cells, establishing a safe therapeutic window for antiviral evaluation.

**Fig. 5:**
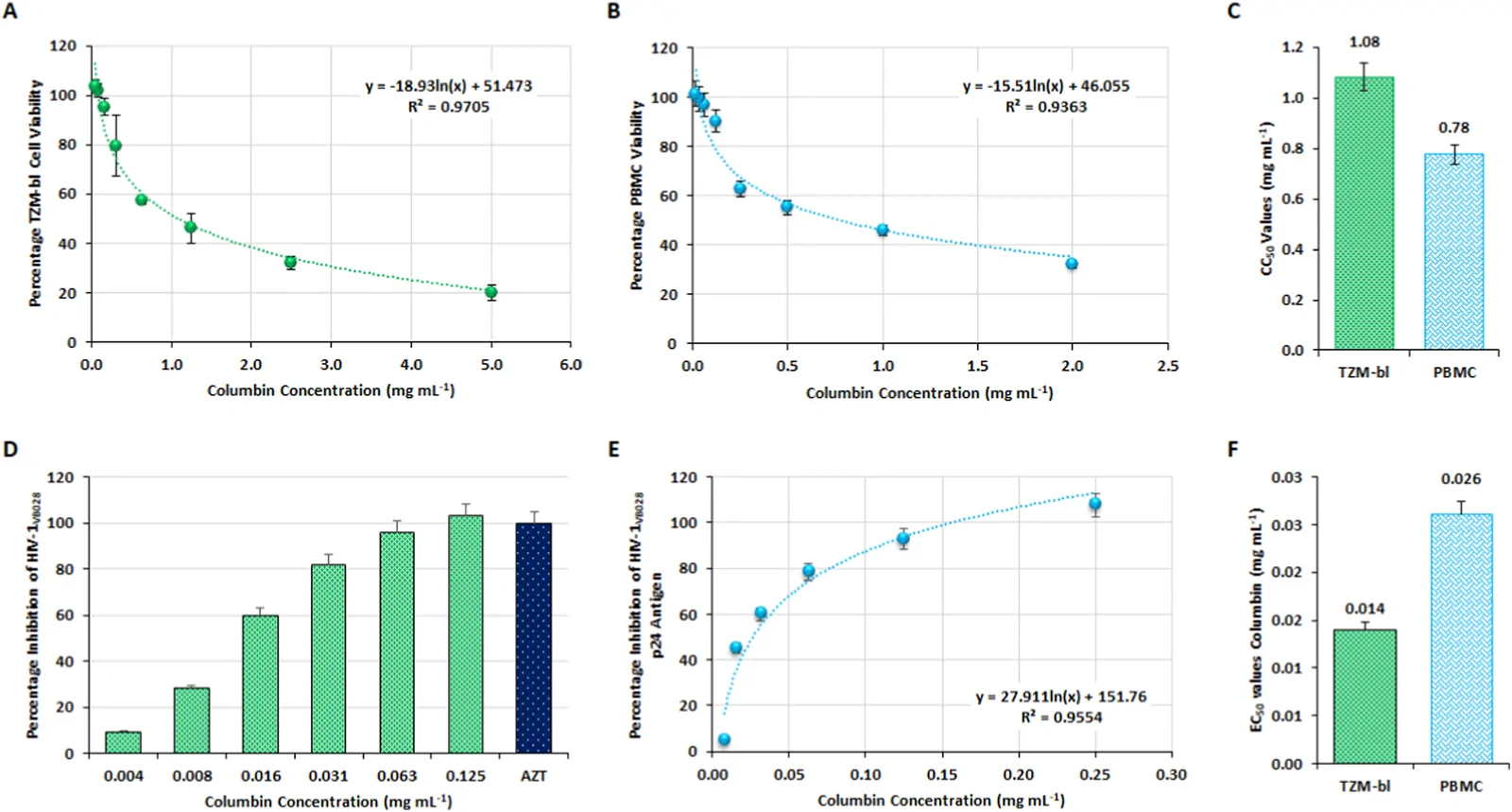
Cytotoxicity and anti-HIV-1 activity of columbin. (A) Dose-dependent cytotoxicity of columbin in TZM-bl cells showing percentage cell viability. (B) Cytotoxic profile of columbin in primary human PBMCs demonstrating concentration-dependent effects on cell viability. (C) Comparative CC_50_ values of columbin in TZM-bl cells and PBMCs. (D) Dose-dependent inhibition of HIV-1_VB028_ replication in TZM-bl cells by columbin, with Azidothymidine (AZT) as positive control. (E) Anti-HIV-1 activity of columbin in HIV-1_VB028_-infected PBMCs measured by p24 antigen levels. (F) Comparative EC_50_ values of columbin in TZM-bl cells and PBMCs. Data are represented as mean ± SD from three independent experiments.

Columbin demonstrated potent dose-dependent anti-HIV-1 activity against HIV-1_VB028_ in both cellular systems. In TZM-bl cells, the compound achieved an EC_50_ of 0.014 mg/mL, with inhibition escalating from 9% at 0.004 mg/mL to complete viral suppression at 0.125 mg/mL (Fig. 5D–F). Validation studies in HIV-1_VB028_-infected PBMCs confirmed significant antiviral efficacy, yielding an EC_50_ of 0.026 mg/mL, as evidenced by dose-dependent reduction in p24 antigen levels. The inhibition profile in PBMCs showed progressive viral suppression from minimal activity at 0.008 mg/mL to complete inhibition at 0.25 mg/mL (Fig. 5E and F). The comparable efficacy across both cellular systems, coupled with the strong computational binding affinity, establishes columbin as a validated lead phytoconstituent from TC with dual-target anti-HIV-1 potential. The selectivity indices (CC_50_/EC_50_) of 77.1 in TZM-bl cells and 30.0 in PBMCs indicate favorable therapeutic margins, supporting its potential for further preclinical development.

#### 3.4.2 Molecular interactions of tinosporaside & tinosporide with HIV-1 protease and reverse transcriptase

In addition to columbin, two other diterpenoid compounds from TC, tinosporaside (CID_14194109) and tinosporide (CID_167631), also demonstrated notable binding affinities toward HIV-1 protease and reverse transcriptase in molecular docking analyses. Both compounds exhibited strong interactions with critical amino acid residues in the active sites of these essential viral enzymes, with binding energies ranging from −8.46 to −8.98 kcal/mol, suggesting their potential as dual-target inhibitors of HIV-1 replication.

Tinosporaside, a diterpenoid glycoside, exhibits remarkable inhibitory potential against two essential HIV-1 enzymatic targets. Molecular docking studies revealed that tinosporaside demonstrates strong binding affinity toward HIV-1 protease with a binding energy of −8.5 kcal/mol and an inhibition constant of 0.59 μM. The compound forms hydrogen bonds with ASP30 and MET46, which are critical residues involved in the catalytic activity of the protease enzyme. Extensive hydrophobic interactions were observed with multiple residues including ASN25, GLY27, ASP29, VAL32, LYS45, ILE47, GLY48, GLY49, PHE53, and ILE84. Additionally, tinosporaside engages in π-alkyl interactions with ALA28 and ILE50, creating a stable binding environment at the protease active site (Supplementary Fig. 3A).

Against HIV-1 reverse transcriptase, tinosporaside exhibited the highest binding affinity among all tested compounds, with a binding energy of −8.98 kcal/mol and an inhibition constant of 0.26 μM. The strong binding is facilitated by hydrogen bonds with LYS103, LYS101, LEU234, and PRO236. The compound establishes extensive hydrophobic interactions with multiple residues including, LEU100, LYS101, LYS102, VAL106, TYR183, GLY190, LYS220, PHE227, and TRP229. π-alkyl interaction with HIS235 and π-sigma interactions with TYR181 and TYR188, which are known to be crucial residues in the non-nucleoside inhibitor binding pocket of reverse transcriptase, further enhance binding stability (Supplementary Fig. 3B). When compared with FDA-approved drugs, tinosporaside showed substantially better binding energy against HIV-1 protease than Ritonavir and stronger affinity for HIV-1 reverse transcriptase than Zidovudine.^48^

Tinosporide, a bioactive furanolactone compound, also demonstrates promising inhibitory potential when evaluated through computational docking against key HIV-1. Tinosporide showed strong binding affinity toward HIV-1 protease with a binding energy of −8.46 kcal/mol and an inhibition constant of 0.63 μM. The compound established hydrogen bonds with ASP29, ASP30, GLY48, and ILE50, which are critical residues involved in the catalytic function of the protease enzyme. Tinosporide also forms hydrophobic interactions with GLY27, GLY49, THR80, and ILE84, while engaging in π-alkyl interactions with ALA28, VAL32, ILE47, and LEU76, which enhance binding specificity and stability at the protease active site (Supplementary Fig. 4A).

When docked with HIV-1 reverse transcriptase, tinosporide exhibited a binding energy of −8.74 kcal/mol with an inhibition constant of 0.39 μM, indicating strong binding affinity. The compound forms a hydrogen bond with LYS101 and TRP229 alongside establishing hydrophobic interactions with PRO95, TYR183, and TYR188. π-alkyl interactions with LEU100 and LYS102, along with a π-sigma interaction with TYR181, a known residue in the non-nucleoside inhibitor binding pocket of reverse transcriptase, further contribute to binding stability (Supplementary Fig. 4B).

Tinosporide demonstrated balanced binding affinity toward both target enzymes, with similar binding energies for protease (−8.46 kcal/mol) and reverse transcriptase (−8.74 kcal/mol). When compared with FDA-approved antiretrovirals, tinosporide exhibits substantially stronger binding energy against HIV-1 protease than the clinically prescribed protease inhibitor Ritonavir (−4.35 kcal/mol), while also demonstrating better binding affinity for HIV-1 reverse transcriptase than the nucleoside reverse transcriptase inhibitor Zidovudine (−7.23 kcal/mol).^48^

### 3.5 *In vitro* viral enzyme inhibition assays

The mechanistic basis of TC extract-mediated HIV-1 inhibition was investigated through targeted enzyme inhibition assays. Both HATC and AQTC extracts were evaluated for their ability to interfere with three critical viral enzymes essential for HIV-1 replication: integrase, protease, and reverse transcriptase. All experiments were conducted using sub-cytotoxic extract concentrations previously determined to be effective against intact virus. These comprehensive enzyme inhibition assays aimed to elucidate whether TC extracts exhibit multifaceted antiviral properties by simultaneously disrupting multiple stages of the HIV-1 replication cycle, potentially explaining their observed efficacy in cellular models.

#### 3.5.1 HIV-1 protease inhibition by the TC extracts

The TC extracts were evaluated for their ability to inhibit HIV-1 protease activity using a fluorescence-based enzymatic assay. At EC_80_ concentration (0.32 mg/ml), the AQTC demonstrated moderate inhibition of HIV-1 protease, reducing enzyme activity by 52.12% (Fig. 6A). The HATC tested at its EC_80_ concentration (0.35 mg/ml), showed substantial inhibitory effects with 69.6% reduction in protease activity. Ritonavir (10 μM) served as a reference standard in the assay, yielding 72.6% inhibition of HIV-1 protease activity, while Pepstatin (1 mM), the kit-provided inhibitor control employed for assay validation, demonstrated 68.63% inhibition of protease function (Fig. 6A).

**Fig. 6:**
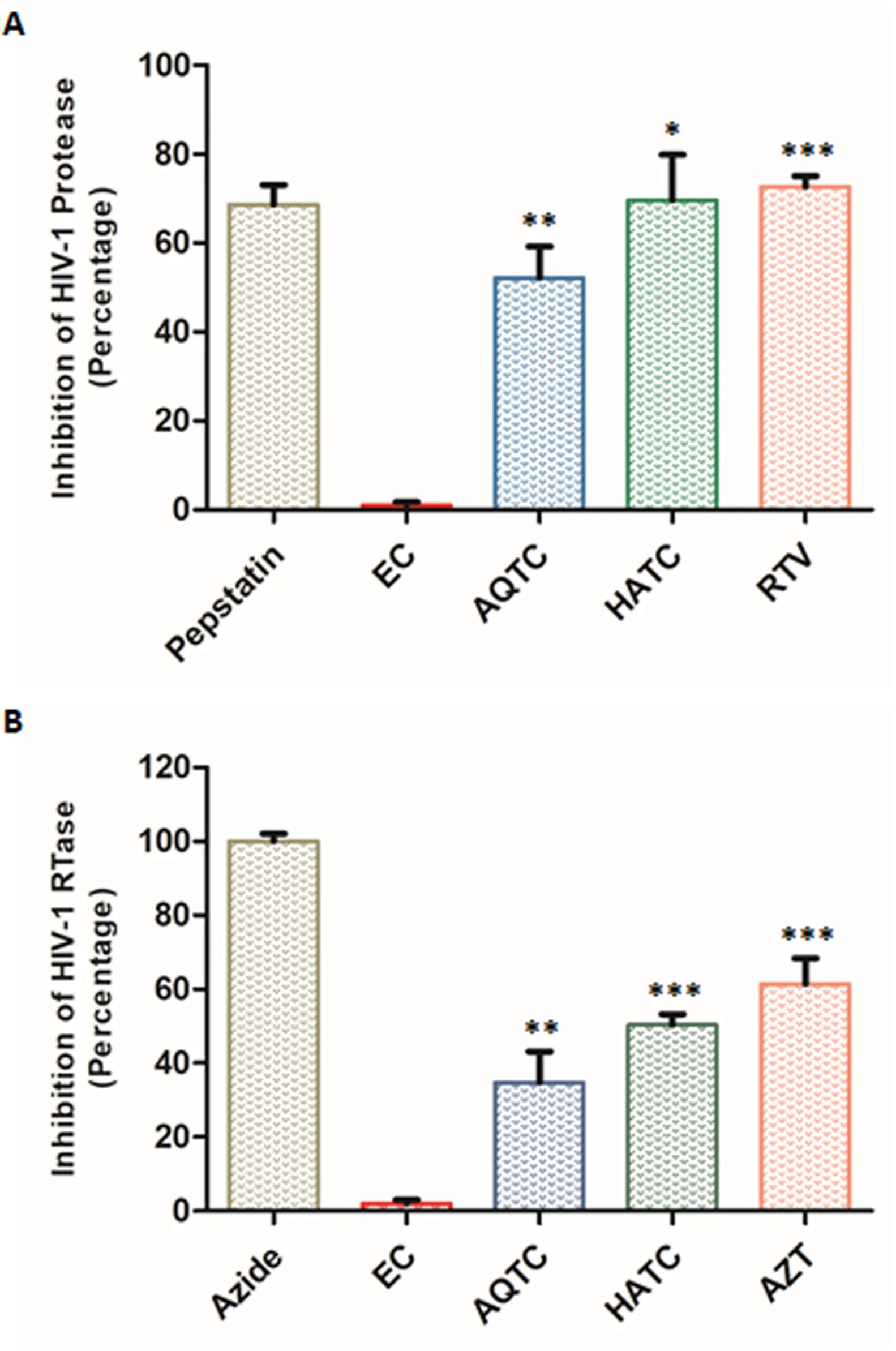
Inhibition of HIV-1 key proteins by *Tinospora cordifolia* aqueous (AQTC) and hydroalcoholic (HATC) extracts. **(A)** The percentage of HIV-1 protease enzyme activity suppression in the presence of AQTC and HATC extracts was compared to the kit provided inhibitor control or positive control (Pepstatin 1 mM), enzyme control (EC), and the known standard drug for HIV-1 protease (Ritonavir 10 μM). **(B)** Percentage inhibition of HIV-1 RTase enzyme activity by AQTC and HATC extracts was examined in comparison to the known HIV-1 RTase inhibitor Azidothymidine (AZT 0.49 μM). To normalize background fluorescence, EC was used as the negative control in each assay following the manufacturers' instructions. The results are shown as the means of at least three experimental replicates with standard deviations represented as the error bar. ∗*p* < 0.05, ∗∗*p* < 0.01, ∗∗∗*p* < 0.001.

#### 3.5.2 *In vitro* inhibition of HIV-1 reverse transcriptase by TC extracts

The TC extracts were assessed for their ability to inhibit HIV-1 reverse transcriptase activity using a previously established protocol.^43^ At EC_80_ concentration (0.35 mg/ml), the HATC demonstrated substantial inhibition of HIV-1 reverse transcriptase, reducing enzyme activity by 50.40% (Fig. 6B). The AQTC tested at the EC_80_ concentration (0.32 mg/ml), showed modest inhibitory effects with a 34.75% reduction in reverse transcriptase activity (Fig. 6B). For comparative evaluation, the standard reverse transcriptase inhibitor AZT was included as a positive control, which exhibited 61.43% inhibition at a 0.3 μM concentration.

#### 3.5.3 Catalytic inhibition of HIV-1 integrase by the extracts

The inhibitory potential of TC extracts against HIV-1 integrase was evaluated through an *in vitro* assay. At EC_80_ concentration, the HATC demonstrated relatively weak inhibition of HIV-1 integrase, reducing enzyme activity by only 30.07%, whereas the AQTC showed suboptimal inhibitory effects with just 23.36% reduction in integrase activity (Supplementary Fig. 5). In contrast, raltegravir (0.48 μM), a clinically approved HIV-1 integrase inhibitor, served as a reference standard, yielding robust 86.76% inhibition of enzyme function, while sodium azide (1%), employed as the positive control for assay validation, demonstrated significant 88.11% inhibition of integrase activity.

### 3.6 HATC treatment modulates cytokine rebalancing and may enhance antiviral responses in HIV-1-infected macrophage

During HIV-1 infection, macrophages mount a strong inflammatory response characterized by the production of multiple inflammatory cytokines. To investigate the immunomodulatory effects of HATC, we evaluated the expression profiles of key inflammatory mediators in HIV-1-infected macrophages (Fig. 7A–I). Differentiated THP-1 macrophages (1 × 10^6^ cells per well) were infected with HIV-1_VB028_ at a TCID_50_ of 400, followed by HATC extract addition at EC_80_ concentration (0.35 mg/ml) to the infected cells, and the assay was terminated at specified time points up to 48 hpi. Results demonstrated that HATC rebalanced the immune response, suggesting a multifaceted role in maintaining cellular homeostasis during HIV-1 infection.

**Fig. 7:**
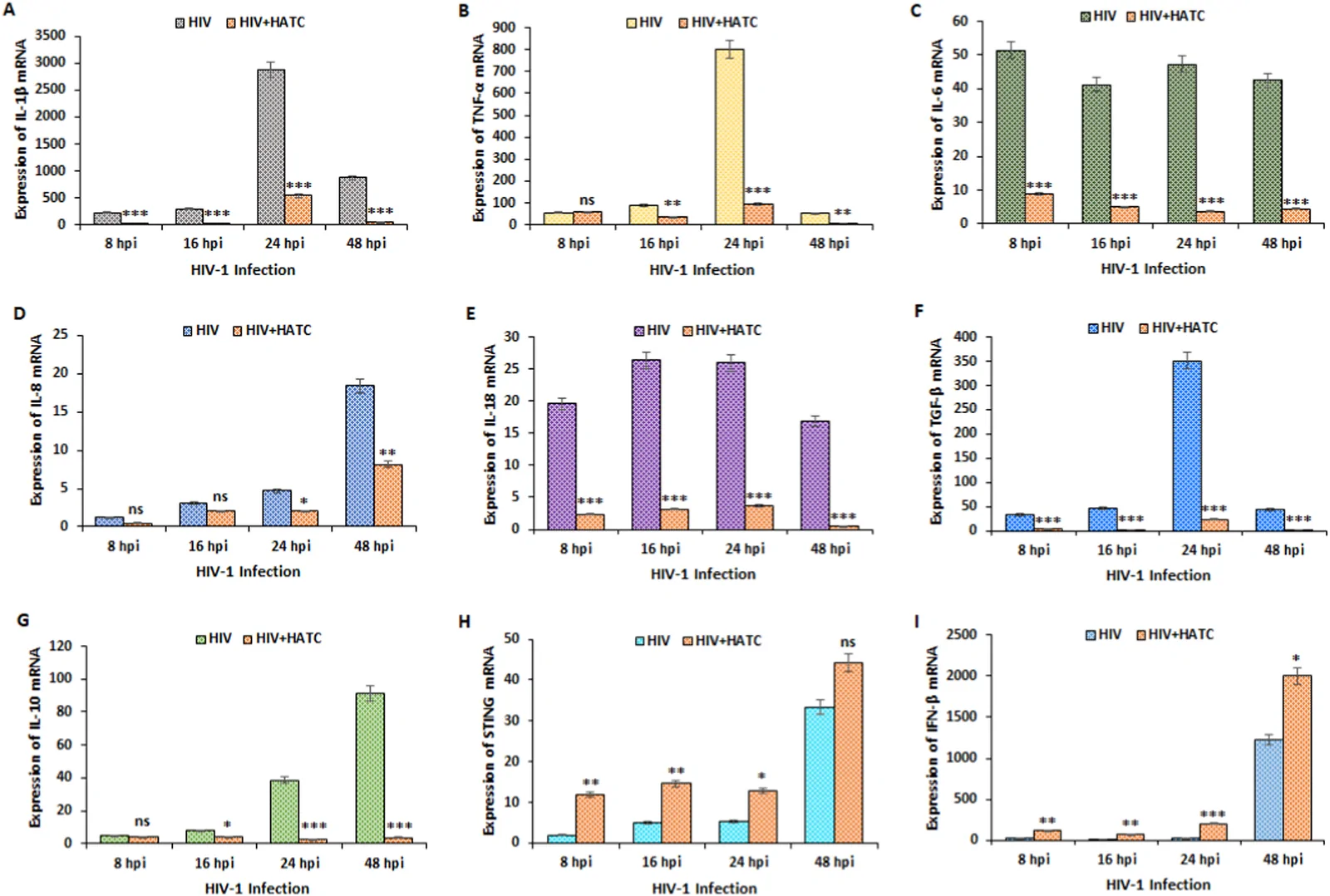
Immunomodulatory effects of *Tinospora cordifolia* hydroalcoholic extract (HATC) on cytokine and innate immune gene expression during HIV-1 infection. Time-course analysis of relative mRNA expression levels of key inflammatory, regulatory, and antiviral genes in HIV-1-infected THP-1-derived macrophages treated with HATC. Gene expression was quantified by qRT-PCR at multiple time points post-infection (8, 16, 24, and 48 h) and normalized to GAPDH. The expression levels are further presented as relative expression compared with uninfected controls. Panels show transcriptional profiles of **(A)** IL-1β, **(B)** TNF-α, **(C)** IL-6, **(D)** IL-8, and **(E)** IL-18 representing pro-inflammatory cytokines; **(F)** TGF-β and **(G)** IL-10 representing immunosuppressive cytokines; and **(H)** STING and **(I)** IFN-β representing key mediators of innate antiviral signalling. HATC treatment markedly attenuated pro-inflammatory and regulatory cytokine responses while enhancing STING and IFN-β expression, indicating a rebalancing of the immune response and activation of host antiviral defence. Data are represented as mean ± SD from three independent experiments. Statistical significance was assessed by one-way ANOVA (∗*p* < 0.05, ∗∗*p* < 0.01, ∗∗∗*p* < 0.001) compared to HIV-infected untreated controls.

#### 3.6.1 HATC treatment suppresses pro-inflammatory cytokine induction in HIV-1-infected macrophages

HIV-1 infection elicits a potent pro-inflammatory response in macrophages, characterized by the overproduction of cytokines.^52^ While these mediators are essential for initiating antiviral defences, their excessive and sustained expression can lead to chronic inflammation, immune exhaustion, and tissue damage. To assess the anti-inflammatory potential of HATC, we examined the effects of pro-inflammatory cytokines, including IL-1β, TNF-α, IL-6, IL-8, and IL-18, in HIV-1-infected macrophages over a 48-h period.

In untreated HIV-1-infected macrophages, IL-1β expression demonstrated a sharp time-dependent increase, peaking at 24 hpi with a 2886-fold elevation compared to uninfected controls. Although levels declined by 48 hpi, they remained significantly elevated, indicating a prolonged pro-inflammatory state. Treatment with HATC significantly suppressed this pro-inflammatory response, reducing it by over 80% at 24 hpi and over 93% at 48 hpi (Fig. 7A). The suppression was maintained across all timepoints, indicating sustained anti-IL-1β activity of HATC throughout the course of infection. Likewise, the TNF-α expression followed a similar temporal dynamic, with substantial upregulation in HIV-1-infected macrophages, reaching its maximum at 24 hpi (approximately 803-fold). However, unlike sustained inflammatory markers, TNF-α levels declined substantially by 48 hpi, revealing a rapid but transient expression pattern. HATC treatment significantly reduced TNF-α expression at both 24 and 48 hpi, with a particularly notable reduction of over 90% by 48 hpi (Fig. 7B).

The IL-6 expression was strongly induced early during HIV-1 infection, with the highest level detected at 8 hpi (51.5-fold increase). While a slight reduction was observed at later timepoints, IL-6 levels remained consistently elevated, indicating a sustained inflammatory response. HATC treatment markedly suppressed IL-6 expression across all timepoints, maintaining levels close to baseline (Fig. 7C), suggesting effective inhibition of IL-6-mediated inflammation throughout the course of infection. In contrast, IL-8 demonstrated a progressive increase during infection that intensified over time, reaching its maximum elevation at 48 hpi (18.4-fold increase). The pattern suggested an escalating inflammatory cascade as infection progressed. HATC treatment substantially attenuated IL-8 expression, with levels reduced by more than 50% at 48 hpi (Fig. 7D).

In addition, IL-18 expression remained elevated throughout HIV-1 infection, with peak levels observed at 16 and 24 hpi (∼26-fold increase). While slightly reduced at 48 hpi, IL-18 levels in untreated macrophages were still significantly above baseline levels. HATC treatment consistently suppressed IL-18 expression at all timepoints, with particularly strong inhibition at 48 hpi, where levels were reduced by over 97% (Fig. 7E). This sustained downregulation indicates that HATC may limit acute inflammatory surges.

Together, these results indicated that HATC effectively suppresses a broad spectrum of pro-inflammatory cytokines induced by HIV-1, albeit with distinct temporal patterns, potentially limiting inflammation-associated tissue damage and immune dysregulation.

#### 3.6.2 HATC treatment restores immune balance by reducing TGF-β and IL-10 in HIV-1-infected macrophages

Chronic HIV-1 infection is often characterized by dysregulation of regulatory cytokines, such as TGF-β and IL-10, which contribute to immune suppression, tissue fibrosis, and viral persistence.^53^^,^^54^ These cytokines play dual roles in immune regulation and inflammation control, but their sustained overexpression during infection can facilitate viral immune evasion and impair antiviral responses. To determine whether HATC modulates this aspect of the host immune response, we assessed the expression profiles of TGF-β and IL-10 in HIV-1-infected macrophages.

TGF-β expression was elevated in untreated HIV-1-infected macrophages, with a pronounced peak at 24 hpi, showing an approximately 350-fold increase relative to uninfected controls. But the expression then dropped back to moderate levels by 48 hpi. The HATC treatment intensely reduced TGF-β expression across all timepoints with decreases exceeding 90% at 16, 24, and 48 hpi (Fig. 7F). This consistent suppression suggests that HATC may mitigate the immunosuppressive signalling and other related effects associated with heightened TGF-β expression during HIV-1 infection.

In contrast, the anti-inflammatory cytokine IL-10 showed a progressive upregulation with levels rising steadily from 8 to 48 hpi, and peaking at a 91-fold induction in untreated infected macrophages. The pattern reflected the establishment of a gradually intensifying immunosuppressive microenvironment. Treatment with HATC effectively normalized IL-10 expression across all timepoints, maintaining levels close to those seen in uninfected controls. Reductions at 24 and 48 hpi were particularly significant, exceeding 90% (Fig. 7G). The normalization of IL-10 expression by HATC suggests that it may help prevent the establishment of an immunosuppressive microenvironment that can hinder effective viral clearance and promote chronic infection.

#### 3.6.3 HATC potentiates early innate immune sensing *via* the STING pathway

Effective control of HIV-1 requires not only suppression of excessive inflammation but also timely activation of antiviral defences.^55^^,^^56^ To determine whether HATC supports innate immune sensing and antiviral signalling, we analyzed the expression of IFN-β and the stimulator of interferon genes (STING) pathway, which is key mediators of type I interferon responses.

In untreated HIV-1-infected macrophages, STING expression exhibited a gradual elevation showing only modest induction in HIV-infected macrophages over time, suggesting limited activation of cytosolic DNA sensing pathways. HATC treatment, however, triggered an early and sustained upregulation of STING expression, starting at 8 hpi and maintained through 48 hpi (Fig. 7H). This early enhancement suggests that HATC may accelerate the initiation of innate immune signalling through the STING pathway.

Consistent with this, IFN-β expression was significantly elevated in HATC-treated infected macrophages across all the time points tested, reaching its highest level at 48 hpi (∼2005-fold) (Fig. 7I), while IFN-β expression in untreated cells remained comparatively low during early infection stages, with a pronounced but delayed induction observed only at 48 hpi (∼1231-fold). This pattern indicated a suboptimal innate antiviral defence mechanism in untreated cells, which was significantly enhanced by the HATC treatment.

Collectively, these findings suggest that HATC treatment may orchestrate a comprehensive rebalancing of the immune microenvironment in HIV-1-infected macrophages by simultaneously attenuating excessive pro-inflammatory signals, normalizing regulatory cytokine responses, and enhancing innate antiviral defence mechanisms. This multifaceted immunomodulatory activity may create a more favorable environment for controlling HIV-1 infection while limiting inflammatory tissue damage.

### 3.7 HATC inhibits HIV-1 gene expression and viral protein production in macrophages

To assess the antiviral efficacy of HATC, we monitored the expression levels of two key HIV-1 genes: GAG (Group-specific Antigen), which plays a central role in the viral life cycle, and TAT (Trans-Activator of Transcription), a regulatory protein implicated in the development of various HIV-associated complications. mRNA expression was analyzed in THP-1-derived macrophages at multiple time points post-infection (8, 16, 24, and 48 h).

In untreated infected cells, GAG mRNA expression increased progressively, reaching a peak at 48 hpi with a 1583.35-fold relative expression (Fig. 8A). Treatment with HATC at 0.35 mg/ml (corresponding to the EC_80_ concentration) resulted in a robust and sustained suppression of GAG expression at all time points, with inhibition ranging from approximately 60% to 97% compared to untreated controls. This reduction was statistically significant from the early stage of infection (8 hpi, *p* < 0.05) and became highly significant at later time points (24–48 hpi, *p* < 0.001). In contrast, TAT mRNA expression in untreated infected macrophages remained consistently elevated throughout the infection period without a progressive temporal pattern (Fig. 8B). HATC treatment led to a marked reduction in TAT expression across all time points, with the most pronounced effect observed at 16 hpi (∼13.4-fold decrease), followed by sustained suppression at 48 hpi (∼7.8-fold) and 24 hpi (∼5.4-fold). These reductions were statistically significant (*p* < 0.01 to *p* < 0.001) at all time points except 8 hpi, where the difference did not reach significance.

**Fig. 8:**
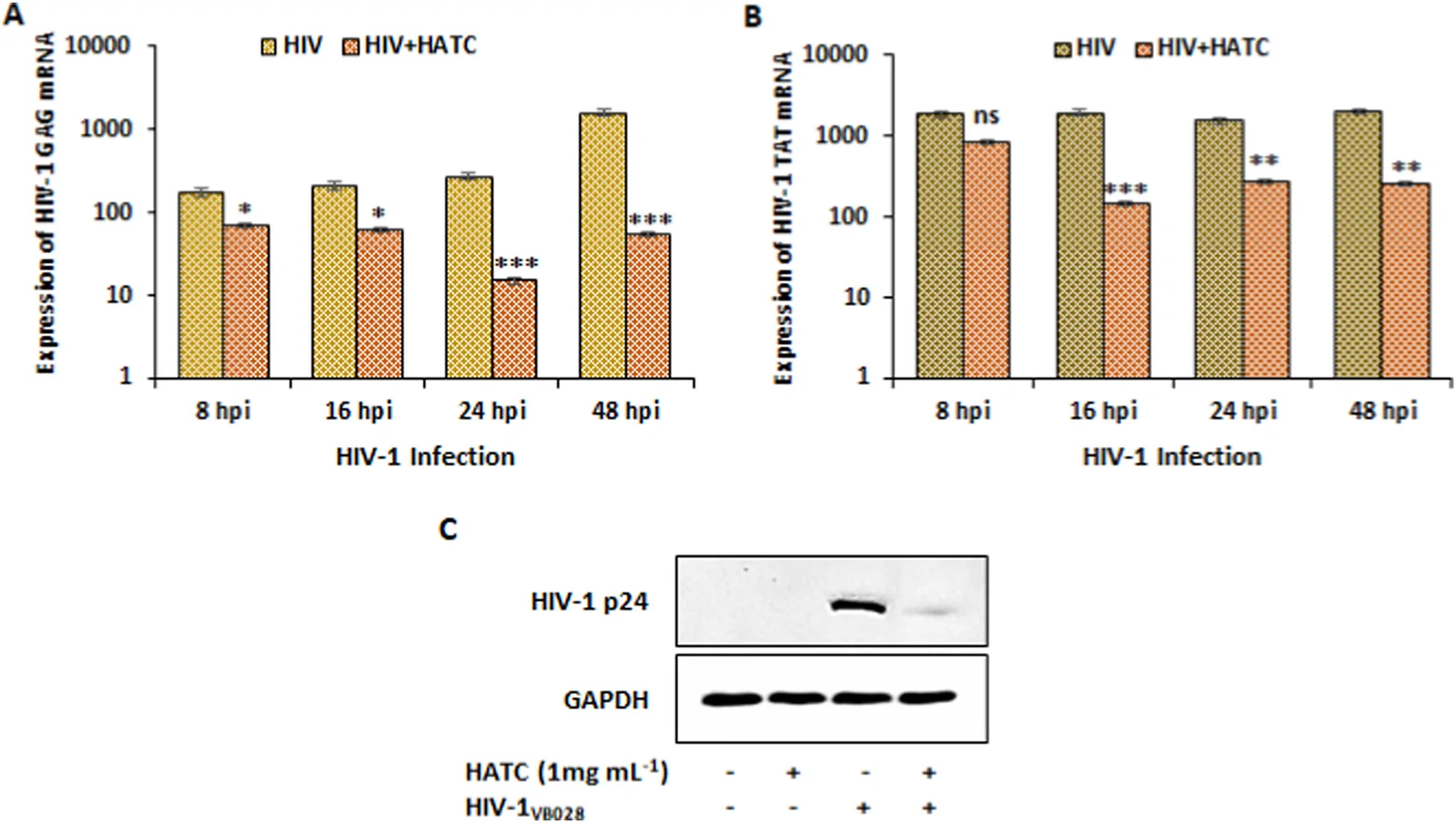
Effect of *Tinospora cordifolia* hydroalcoholic extract (HATC) on HIV-1 infection. Relative expression of **(A)** HIV-1 GAG and **(B)** TAT gene in presence of EC_80_ concentrations (0.35 mg/ml) of HATC treatments in HIV-1_VB028_ infected cells. **(C)** Level of HIV-1 p24 antigen in presence of sub-cytotoxic concentration of HATC treatments in HIV-1_VB028_ infected cells. The results shown as the means of at least three experimental replicates plus the standard deviations were calculated and represented as the error bar. ∗*p* ≤< 0.05, ∗∗*p* ≤ <0.01, ∗∗∗*p* < 0.001.

To validate these transcriptional findings at the protein level, we performed immunoblot analysis for the HIV-1 p24 capsid protein. Consistent with the mRNA data, p24 protein levels were significantly reduced in HATC-treated cells compared to untreated infected controls (Fig. 8C). Collectively, these results demonstrate that HATC exerts a potent antiviral effect against HIV-1 in THP-1-derived macrophages by significantly suppressing viral gene expression at both the transcriptional and translational levels.

## 4 Discussion

This study presents the comprehensive evidence of the anti-HIV-1 potential of TC extracts, particularly highlighting the hydroalcoholic extract as a promising candidate with multi-targeted antiviral activity. Although prior studies have explored TC in the context of HIV, they were limited to clinical observations in HIV-positive patients and preliminary enzyme inhibitory activity of leaf extracts, without demonstrated viral inhibition in infected cellular models.^41^^,^^42^ The present study advances this understanding through a broad spectrum of assays encompassing direct viral inhibition against primary HIV-1 isolates of distinct subtypes, simultaneous targeting of all three key viral enzymes, phytoconstituent identification and experimental validation, and immunomodulatory characterization in HIV-1-infected macrophages, collectively supporting the traditional use of TC in Ayurvedic medicine and positioning it as a scientifically grounded candidate for adjunctive HIV-1 therapy.

Initial screening of both the aqueous and hydroalcoholic extracts revealed significant anti-HIV-1 activity in cell-based assays (Fig. 2, Fig. 3). Both extracts inhibited HIV-1 infection in TZM-bl cells and PBMCs, with acceptable selectivity indices observed in the physiologically relevant primary cell model. To further substantiate the antiviral potential under increased infectious pressure, both TC extracts and columbin were evaluated against HIV-1_VB028_ infection using a 10-fold higher viral inoculum (400 TCID_50_) in both TZM-bl cells and PBMCs (the latter seeded at 2 × 10^4^ cells/well). Under these conditions, AQTC, HATC, and columbin maintained dose-dependent antiviral activity in TZM-bl cells with EC_50_ values of 0.084 mg/mL, 0.213 mg/mL, and 0.095 mg/mL, respectively, and in PBMCs with EC_50_ values of 0.139 mg/mL, 0.188 mg/mL, and 0.045 mg/mL, respectively (Supplementary Fig. 6), confirming their strong antiviral efficacy even against high viral loads in both cell line and primary human cells. Particularly, the HATC consistently exhibited lower EC_50_ values, stronger inhibition in both cell-associated and cell-free conditions, and higher potency in primary cells (Fig. 3 and Supplementary Fig. 6). Notably, while AQTC demonstrated some efficacy, its activity was less pronounced across assays and enzyme inhibition studies. For example, HATC inhibited HIV-1 protease and reverse transcriptase by 69.6% and 50.4%, respectively, compared to 52.1% and 34.8% inhibition by AQTC (Fig. 6). Given its superior potency and broader activity, we advanced HATC for in-depth mechanistic and immunological evaluation.

A central observation was that HATC directly interferes with HIV-1 replication at the genetic level. Quantitative PCR and immunoblot analyses in THP-1-derived macrophages showed significant suppression of GAG and TAT mRNA expression (Fig. 8A and B), as well as reduced levels of the viral p24 protein (Fig. 8C). These data confirm that HATC impedes both the transcription and translation of viral components essential for replication and pathogenesis. Given the role of TAT in immune activation and viral transcription elongation 57, 58, 59, 60, the downregulation of TAT highlights a potentially valuable mechanism through which HATC might limit HIV-associated inflammation and latency establishment.

The robust antiviral effects observed at the cellular and molecular levels prompted us to examine the immunological consequences of HATC treatment in HIV-1-infected macrophages. While TC has been documented for its immunomodulatory effects in non-HIV models or clinical settings, its precise mechanism and immune regulatory role in the context of HIV infection have not been established.^61^^,^^62^ Chronic immune activation is a hallmark of HIV-1 pathogenesis and is primarily driven by persistent pro-inflammatory cytokine release and dysregulation of regulatory mediators.^56^^,^^63^^,^^64^ Our data showed that untreated infected macrophages exhibited sharp increases in key pro-inflammatory cytokines like IL-1β, TNF-α, IL-6, IL-8, and IL-18, throughout the 48-h infection period (Fig. 7A–E). Notably, IL-1β levels surged over 2800-fold at 24 hpi (Fig. 7A), and TNF-α rose ∼800-fold (Fig. 7B), reflecting a cytokine storm-like profile associated with tissue damage, T cell exhaustion, and viral persistence, as reported in earlier studies.^52^^,^^53^

HATC treatment dramatically suppressed these cytokine surges, reducing IL-1β and TNF-α by over 80–90% at their peak expression times, and bringing IL-6, IL-8, and IL-18 closer to basal levels (Fig. 7A–E). This indicates a potent anti-inflammatory capability, likely mediated by the phytoconstituents’ ability to modulate upstream signalling pathways such as NF-κB or inflammasome activation^65^, ^66^, ^67^, ^68^. Such suppression of early inflammatory mediators can reduce immune exhaustion and preserve tissue integrity during HIV infection, supporting a more favorable immune landscape for viral control^64^^,^^69^, ^70^, ^71^.

In addition to controlling inflammation, HATC normalized the expression of regulatory cytokines such as TGF-β and IL-10 (Fig. 7F and G). In untreated infected macrophages, both cytokines were upregulated, TGF-β by nearly 350-fold and IL-10 by over 90-fold, suggesting a compensatory immune suppressive response that can ultimately favour viral persistence and latency.^54^ HATC markedly reduced their expression across all time points, particularly at 24 and 48 hpi, suggesting a role in restoring immune homeostasis and limiting the immunosuppressive niche that HIV exploits to evade clearance.

Of particular importance is the finding that HATC may enhance innate antiviral signalling via the STING–IFN-β pathway. STING expression in untreated cells remained low throughout the infection course (Fig. 7H), indicating inadequate cytosolic DNA sensing, a common impairment in HIV-infected macrophages.^55^ HATC treatment induced a rapid and sustained upregulation of STING from 8 hpi onward, along with a parallel increase in IFN-β, reaching over 2005-fold induction by 48 hpi (Fig. 7I). This suggests that HATC not only suppresses immune dysregulation but also actively primes innate defences against HIV-1. Given that early and strong type I interferon responses are crucial for restricting HIV replication and reservoir formation,^56^ this upregulation represents a key mechanism by which HATC may exert long-term protective effects.

Interestingly, treatment with HATC induced a selective pattern of STING pathway activation. While canonical STING activation is expected to drive both IRF3-mediated type I interferon responses and NF-κB–driven pro-inflammatory cytokines, HATC upregulated IFN-β while concurrently suppressing IL-1β and TNF-α. Notably, passive cytokine reduction resulting from decreased viral load would manifest as uniform immune dampening rather than the observed selective enhancement of antiviral interferon signalling. This suggests a biased activation of STING signaling, favoring antiviral interferon responses while minimizing inflammatory cytokine output. Such uncoupling of STING's dual functions has been reported in earlier studies, supporting the plausibility of our findings^72^, ^73^, ^74^. At the same time, we acknowledge that the extract represents a complex mixture of phytoconstituents, and the observed pattern likely reflects a composite outcome of multiple compounds acting synergistically or antagonistically. The precise molecular interactions underlying this bias were not dissected in the present study, and future investigations should explore the in-depth interplay between NF-κB and STING signalling pathways in this context to elucidate the mechanistic basis of this selective activation. Also, it is important to consider that our infection model spans a 48-h period, and while these findings demonstrate beneficial immune modulation during the acute phase of infection, the long-term consequences of STING pathway activation in chronic HIV infection cannot be inferred from the current dataset and require future investigation. Nevertheless, this study provides a critical foundation for understanding HATC's immunomodulatory potential in HIV-1 infection. Additionally, we acknowledge that the present study did not include functional assessments of macrophage polarization states, specifically M1 (pro-inflammatory) versus M2 (anti-inflammatory/regulatory) phenotypes. Macrophage polarization is a critical determinant of immune outcomes in HIV infection, and characterization of M1/M2 markers (such as CD80/CD86, CD163/CD206, iNOS, and Arginase-1) would provide deeper mechanistic insight into how HATC influences cellular immune function. The absence of these phenotypic markers represents a limitation of our study, and future investigations should incorporate immunophenotyping to validate whether the observed cytokine changes correlate with shifts in macrophage polarization and functional status.

The integration of molecular docking and viral enzyme assays further supports the dual-action model of HATC activity. Phytochemicals such as columbin, tinosporide, and tinosporaside demonstrated strong binding affinities and inhibitory potential against HIV-1 protease and reverse transcriptase (Supplementary Tables 2 and 3; Fig. 4, Supplementary Figs. 3 and 4). These compounds interact with catalytically critical residues via hydrogen bonding and hydrophobic interactions, thereby blocking enzymatic function and potentially halting viral maturation and reverse transcription. The poor docking and weak inhibitory profile against integrase are consistent across compounds and suggest that the primary antiviral mechanisms of HATC target earlier phases of the viral lifecycle. However, it is important to note that molecular docking provides computational predictions of binding affinity and inhibition constants (Ki) under idealized conditions, whereas *in vitro* enzyme inhibition depends on multiple factors including experimental conditions, enzyme kinetics, compound solubility, stability, conformational flexibility, bioavailability in the assay system, synergistic effects of multiple compounds, and molecular interactions beyond the active site.^75^ These computational parameters thus serve as relative indicators rather than absolute predictors of biological activity. Although columbin demonstrated a stronger predicted binding energy (−9.04 kcal/mol) than Ritonavir (−4.35 kcal/mol) against HIV-1 protease, the hydroalcoholic extract exhibited slightly lower inhibition rate *in vitro* may be attributed to such pharmacokinetic and physicochemical limitations, including the unknown and potentially low effective concentration of columbin within the extract. Ritonavir, as a clinically optimized protease inhibitor, exhibits high assay-specific efficacy due to extensive pharmaceutical development, despite showing a comparatively weaker docking score. This disparity underscores the importance of integrating computational predictions with experimental validation and highlights that strong binding affinity alone does not guarantee superior biological activity. To validate these computational predictions, commercially available columbin, which exhibited the strongest predicted binding affinity against HIV-1 protease, was evaluated for antiviral efficacy in cellular models. Columbin demonstrated potent dose-dependent anti-HIV-1 activity in both TZM-bl cells and PBMCs (Fig. 5), with favorable selectivity indices, supporting its predicted antiviral potential. While molecular docking studies identified several promising phytoconstituents with predicted anti-HIV-1 activity, only columbin was experimentally validated through cell-based antiviral assays in this study. Several identified compounds, perticulary tinosporide and tinosporaside, despite exhibiting strong computational binding affinities, were not tested in isolation against HIV-1 in cell-based or enzyme inhibition assays in this study. This represents a limitation of our work, as it prevents comprehensive assessment of individual compound potencies, bioavailability, and potential synergistic or antagonistic interactions. Future studies should prioritize the isolation, purification, and individual evaluation of these key phytochemicals to facilitate rational drug design for HIV-1 therapy.

In summary, this study underscores the therapeutic potential of TC, particularly its hydroalcoholic stem extract, as a multi-target inhibitor of HIV-1. HATC effectively suppresses viral replication, inhibits critical viral enzymes, attenuates pro-inflammatory cytokine expression, normalizes regulatory cytokines, and promotes antiviral innate immunity via the STING–IFN-β axis. Columbin, one of the bioactive phytoconstituents from the extract, demonstrated potent antiviral activity against HIV-1 in cellular models. These multifaceted effects are especially valuable in the context of chronic HIV infection, where immune dysfunction and drug resistance pose significant clinical challenges. While these results are promising, future work should focus on *in vivo* validation, pharmacokinetic profiling, and synergy studies with standard antiretroviral drugs.

## 5 Conclusion

This study provides the first integrated virological, biochemical, molecular, and immunological characterization of TC extracts, identifying the hydroalcoholic extract as a promising multi-target antiviral agent capable of inhibiting HIV-1 replication through both direct and immunomodulatory mechanisms. HATC significantly suppressed viral gene expression and protein production, inhibited reverse transcriptase and protease activities, and may have enhanced innate antiviral responses via STING–IFN-β signalling, while simultaneously attenuating pro-inflammatory and regulatory cytokines in HIV-1-infected macrophages. Molecular docking supported the involvement of key phytochemicals, columbin, tinosporide, and tinosporaside, in targeting viral enzymes. Experimental validation of columbin confirmed its potent anti-HIV-1 activity in both TZM-bl cells and PBMCs, supporting the computational predictions. While promising, the study is limited to *in vitro* evaluations, and further studies on pre-clinical trials involving PLHIV (People living with HIV) patients are essential to validate efficacy, bioavailability, and safety. Future work should focus on isolating key active constituents, exploring formulation strategies, and assessing synergy with existing antiretrovirals. Overall, our findings highlight *Tinospora cordifolia*, a widely used Ayurvedic Rasayana herb, as a promising phytotherapeutic candidate for HIV-1 intervention, offering a dual approach of viral inhibition and immune restoration.

## Ethics approval and consent to participate

The study was approved by the Institutional Ethics Committee of ICMR *vide* Protocol No.: NARI/EC/Approval/2021-15/539, dated 03/11/2021. Informed consent was not applicable, as the study did not involve human participants or the use of identifiable human specimens.

## Availability of data and materials

All data generated or analyzed during this study are included in this published article and its supplementary information files.

## Consent for publication

Not applicable. The manuscript does not contain data from any individual person.

## Author contributions

Conceptualization, R.H. and A.M.; methodology, R.H., P.J., C.S., S.Y.G., As.M., and M.C.T.; software, R.H., and C.S.; validation, R.H., C.S., P.J., and A.M.; formal analysis, R.H., P.J., and A.M.; investigation, R.H., P.J., S.Y.G., and C.S.; resources, S.B., P.C.G., G.T., and A.M.; data curation, R.H., C.S., P.J., and A.M.; writing-original draft preparation, R.H., and A.M.; writing-review and editing, P.J., C.S., A.M.; visualization, R.H., P.J. and A.M.; supervision, A.M.; project administration, P.J. and A.M.; funding acquisition, P.J. and A.M. All authors read and approved the final manuscript.

## Funding

This research was funded by the Department of Health Research (DHR) Women Scientist Grant, vide no. WSS/2020/000023/AP_WSS, Indian Council of Medical Research.

## Declaration of competing interest

The authors declare that they have no competing interests.
