# Supplementary material for: Anti-HIV-1 potential of Tinospora cordifolia extracts: Multi-targeted viral inhibition and immune landscape modulation
Source: J Tradit Complement Med. 2026 Jun 11;16(6):584–98. doi: 10.1016/j.jtcme.2026.06.003 (PMC13729073; doi:10.1016/j.jtcme.2026.06.003)
Supplement: Supplementary file 1 [file mmc1.pdf]

Supplementary Data

Anti-HIV-1 potential of *Tinospora cordifolia* extracts: Multi-targeted viral inhibition and immune landscape modulation

Supplementary Table

Supplementary Table 1: List of primer sequences used in this study.

No.	Oligo Name	5'→3' Sequence
1	IL-1β-F	CCACAGACCTTCCAGGAGAATG
2	IL-1β-R	GTGCAGTTCAGTGATCGTACAGG
3	TNFα-F	CTCTTCTGCCTGCTGCACTTTG
4	TNFα-R	ATGGGCTACAGGCTTGTCACTC
5	IL6-F	AGACAGCCACTCACCTCTTCAG
6	IL6-R	TTCTGCCAGTGCCTCTTTGCTG
7	IL8-F	GAGAGTGATTGAGAGTGGACCAC
8	IL8-R	CACAACCCTCTGCACCCAGTTT
9	IL18-F	GATAGCCAGCCTAGAGGTATGG
10	IL18-R	CCTTGATGTTATCAGGAGGATTCA
11	TGFβ1-F	TACCTGAACCCCGTGTTGCTCTC
12	TGFβ1-R	GTTGCTGAGGTATCGCCAGGAA
13	IL10-F	TCTCCGAGATGCCTTCAGCAGA
14	IL10-R	TCAGACAAGGCTTGGCAACCCA
15	STING-F	CCTGAGTCTCAGAACAACTGCC
16	STING-R	GGTCTTCAAGCTGCCCACAGTA
17	IFNβ1-F	CTTGGATTCCTACAAAGAAGCAGC
18	IFNβ1-R	TCCTCCTTCTGGAACTGCTGCA
19	GAPDH-F	GTCTCCTCTGACTTCAACAGCG
20	GAPDH-R	ACCACCCTGTTGCTGTAGCCAA

Supplementary Table 2: Molecular interactions profile of HIV-1 protease (5KR0) with phytomolecules of *Tinospora cordifolia.*

Sl. No.	Name of molecule	Binding Energy	Inhibition Constant (Ki)	Hydrogen Bonds	Hydrophobic Bonds	π-alkyl Bonds	π-sigma
1	20_Hydroxy_Ecdysone (CID_5459840)	-8.61	500.76	ASN25, ASP29, ASP30, GLY48, ILE50	GLY27, LYS45, ILE47, GLY49, GLY51, GLY52, PHE53, LEU76, THR80	ALA28, VAL32, ILE84	
2	Berberine (CID_2353)	-7.79	1.96	ASP30	ASP29, THR31, GLY48, GLY49, GLY51, GLY52, THR80, PRO81, ILE84	ALA28, VAL32, ILE54, LEU76	ILE47, ILE54
3	Columbamine (CID_72310)	-6.72	11.86	ASP29, ASP30	GLY49, ILE50, GLY51, GLY52, PHE53, LEU76	ALA28, ILE47, GLY48, ILE54, PRO81	
4	Columbin (CID_188289)	-9.04	234.7	ASP29, ASP30, ILE47, GLY48, ILE50	LYS45, GLY52, PHE53, LEU76, THR80, ILE84	ALA28, VAL32, ILE54	
5	Magnoflorine (CID_73337)	-6.73	11.59	ASN25, GLY27, ASP29, ILE47, GLY52	ASP30, GLY48, GLY49, PHE53, ILE54, ILE84	ALA28	
6	Menisperine (CID_161487)	-6.85	9.54	GLY27, ASP29, ILE47, GLY49	LEU23, ASN25, ASP30, GLY48, GLY49, ILE50, PHE53, ILE54	ALA28, ILE84	
7	Syringin (CID_5316860)	-5.28	133.72	GLY27, ASP29, ASP30, GLY48, THR80	ASN25, THR31, LYS45, PHE53, ILE54, PRO81, VAL82	ALA28, VAL32, ILE47, LEU76, ILE84	
8	Tinocordiside (CID_177384)	-7.68	12.78	GLY27, ASP29, ASP30	ASN25, ALA28, GLY48, GLY49, ILE50, GLY52, VAL56, LEU76, THR80, PRO81	VAL32, ILE47, ILE54, ILE84	
9	Tinosporaside (CID_14194109)	-8.5	589.32	ASP30, MET46	ASN25, GLY27, ASP29, VAL32, LYS45, ILE47, GLY48, GLY49, PHE53, ILE84	ALA28, ILE50	
10	Tinosporide (CID_167631)	-8.46	631.3	ASP29, ASP30, GLY48, ILE50	GLY27, GLY49, THR80, ILE84	ALA28, VAL32, ILE47, LEU76	

Supplementary Table 3: Molecular interactions profile of HIV-1 reverse transcriptase (3QO9) with phytomolecules of *Tinospora cordifolia*.

Sl. No.	Name of molecule	Binding Energy	Inhibition Constant (Ki)	Hydrogen Bonds	Hydrophobic Bonds	π-alkyl Bonds	π-sigma
1	20_Hydroxy_Ecdysone (CID_5459840)	-8.26	885.18	LYS103	LYS101, LYS102, LYS104, SER105, TYR181, TYR183, LYS220, PHE227	LEU100, VAL106, TYR188, LEU234	TRP229
2	Berberine (CID_2353)	-8.06	1.25	LYS223	HIS221, GLN222	LEU100, VAL108, TYR188, LYS220, PHE227, TRP229, LEU234	
3	Columbamine (CID_72310)	-7.54	3	HIS221, LYS223	VAL106, LYS220, GLN222	LEU100, TYR188, PHE227, LEU234	VAL108, TRP229
4	Columbin (CID_188289)	-8.28	851.72	LYS101, LYS103	LEU234, PRO236, TYR318	PRO95, LYS102, VAL106, TYR188, TRP229	LEU100, TYR181
5	Magnoflorine (CID_73337)	-7.64	2.51	LYS101, TYR181	PRO95, GLY99, LYS102, GLY190	LYS103, VAL106	LEU100
6	Menisperine (CID_161487)	-7.38	3.9	LYS101	LYS102, LYS103, GLY190, TYR188, PHE227, LEU234	VAL106, VAL179, TYR181	LEU100
7	Syringin (CID_5316860)	-6.08	35.18	VAL106, VAL108, TYR188, LYS223	THR107, LEU109, ASP218, LYS219, HIS221, GLN222, LYS220	PHE227	
8	Tinocordiside (CID_177384)	-8.62	477.56	LYS101, LEU234	LYS102, VAL179, GLY190, PRO225, PRO226, PHE227, PRO236, HIS235	LEU100, LYS103, VAL106, TYR181, TYR188	TYR318
9	Tinosporaside (CID_14194109)	-8.98	261.82	LYS103, LYS101, LEU234, PRO236	LEU100, LYS101, LYS102, VAL106, TYR183, GLY190, LYS220, PHE227, TRP229	HIS235	TYR181, TYR188
10	Tinosporide (CID_167631)	-8.74	394.64	LYS101, TRP229	PRO95, TYR183, TYR188	LEU100, LYS102	TYR181

Supplementary Table 4: Molecular interactions profile of Integrase (1QS4) with phytomolecules of *Tinospora cordifolia.*

Sl. No.	Name of molecule	Binding Energy	Inhibition Constant (Ki)	Hydrogen Bonds	Hydrophobic Bonds	π-alkyl Bonds	π-sigma
1	20_Hydroxy_Ecdysone (CID_5459840)	-5.07	191.51	HIS67, GLU92, SER119, AS155, LYS156	ASP64, CYS65, THR66, ASP116, GLY118, AS120, PHE121, LYS152, LYS159		
2	Berberine (CID_2353)	-6.05	37.05	GL148, LYS159	THR66, CYS65, ASP116, GLY149, ILE151, GLU152, AS155, LYS156	HIS67	ASP64
3	Columbamine (CID_72310)	-5.63	74.64	CYS65, HIS67, GL148, LYS159	ASP64, THR66, GLY149, GLU152, AS155, LYS156		ILE151
4	Columbin (CID_188289)	-6.43	19.32	LYS188, ARG199	MET154, GLU157, LYS160, ARG187, GLY189, TYR194, SER195	HIS183, ALA196	LYS186
5	Magnoflorine (CID_73337)	-6.58	15.15	THR66, AS155, LYS159	CYS65, GLU152	LYS156	ASP64, HIS67, GLU92, ASP116
6	Menisperine (CID_161487)	-6.34	22.56	THR66, AS155, LYS159	CYS65, LEU68, GLU152, LYS156		ASP64, HIS67, GLU92, ASP116
7	Syringin (CID_5316860)	-4.56	459.73	THR66, GLN148, GLU152, LYS159	CYS65, HIS67, ASP116, GLY149, ILE151, ASN155, LYS156		ASP64
8	Tinocordiside (CID_177384)	-4.64	397.34	ASP64, LYS65, HIS67, ASP116, ASN155, LYS159	THR66, GLU152	LYS156	
9	Tinosporaside (CID_14194109)	-6.2	28.45	ASP64, GLU92, ASP116, GLN148, ASN155	HIS67, GLY118, ASN120, PHE121, ILE151, GLU152		
10	Tinosporide (CID_167631)	-5.77	58.99	ASP64, CYS65, ASP116, SER119	THR66, HIS67, THR93, ASN120, GLY118, PHE121		SER119, GLU92

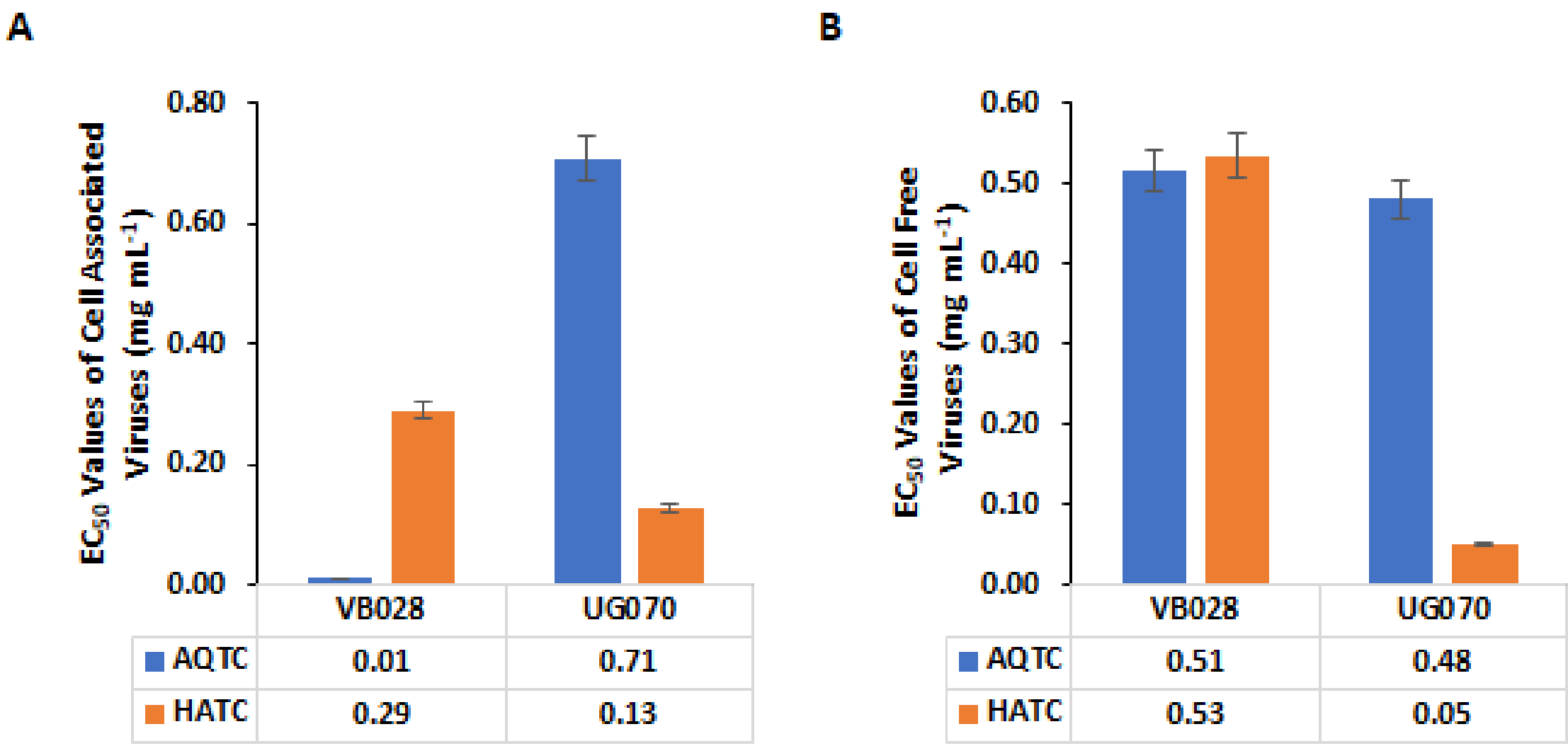

Supplementary Figure 1: Comparison between the EC_{50} values of *Tinospora cordifolia* aqueous extract (AQTC) and *Tinospora cordifolia* hydroalcoholic extract (HATC) in TZM-bl infected with two HIV-1 subtypes in (A) cell-associated and (B) cell-free assays. The results shown are the means of at least three experimental replicates plus the standard deviations were calculated and represented as the error bar.

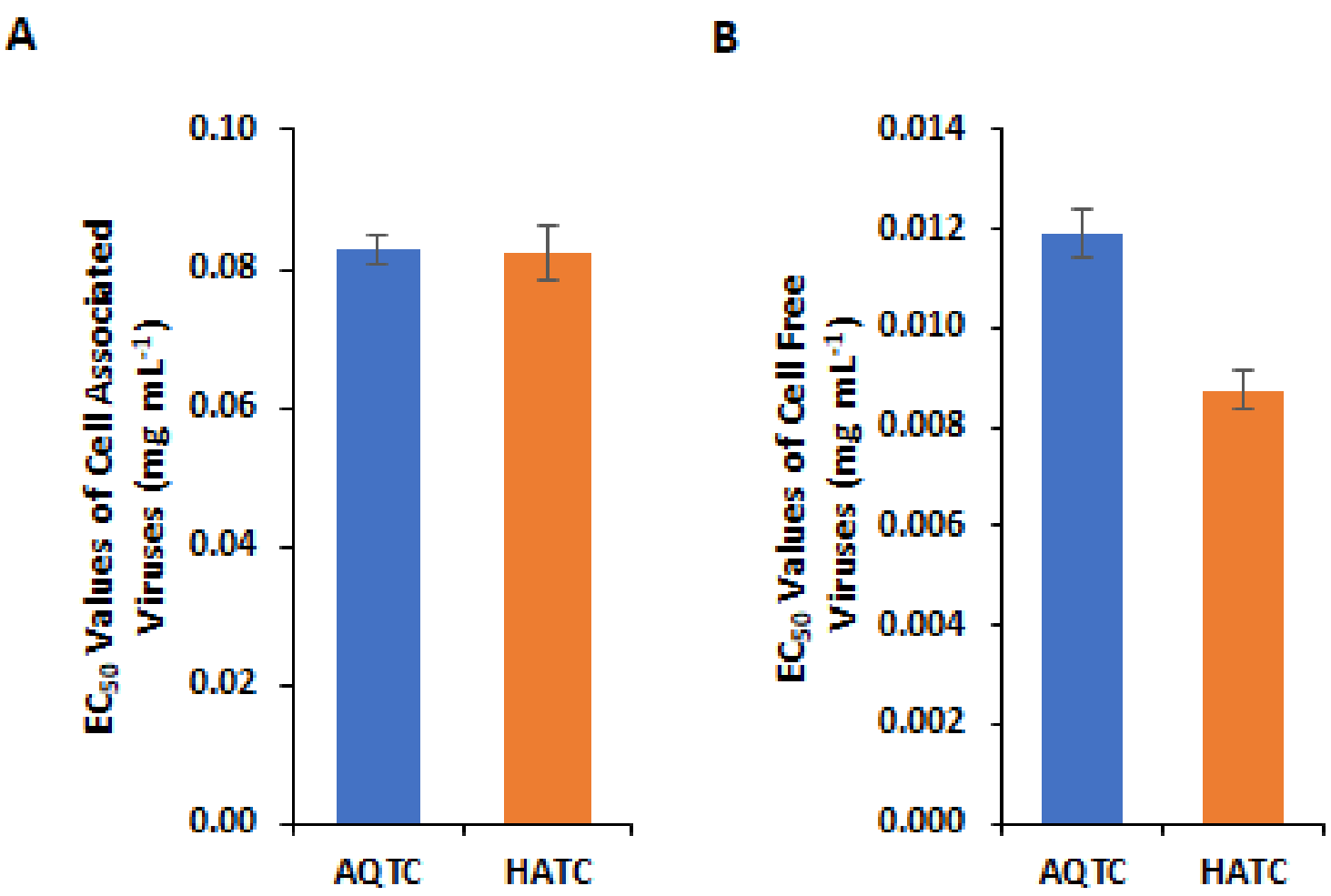

Supplementary Figure 2: Comparison between the EC_{50} values of *Tinospora cordifolia* aqueous extract (AQTC) and T*inospora cordifolia* hydroalcoholic extract (HATC) in PBMCs infected with HIV-1_{VB028} subtype in (A) cell-associated and (B) cell-free assays. The results shown are the means of at least three experimental replicates plus the standard deviations were calculated and represented as the error bar.

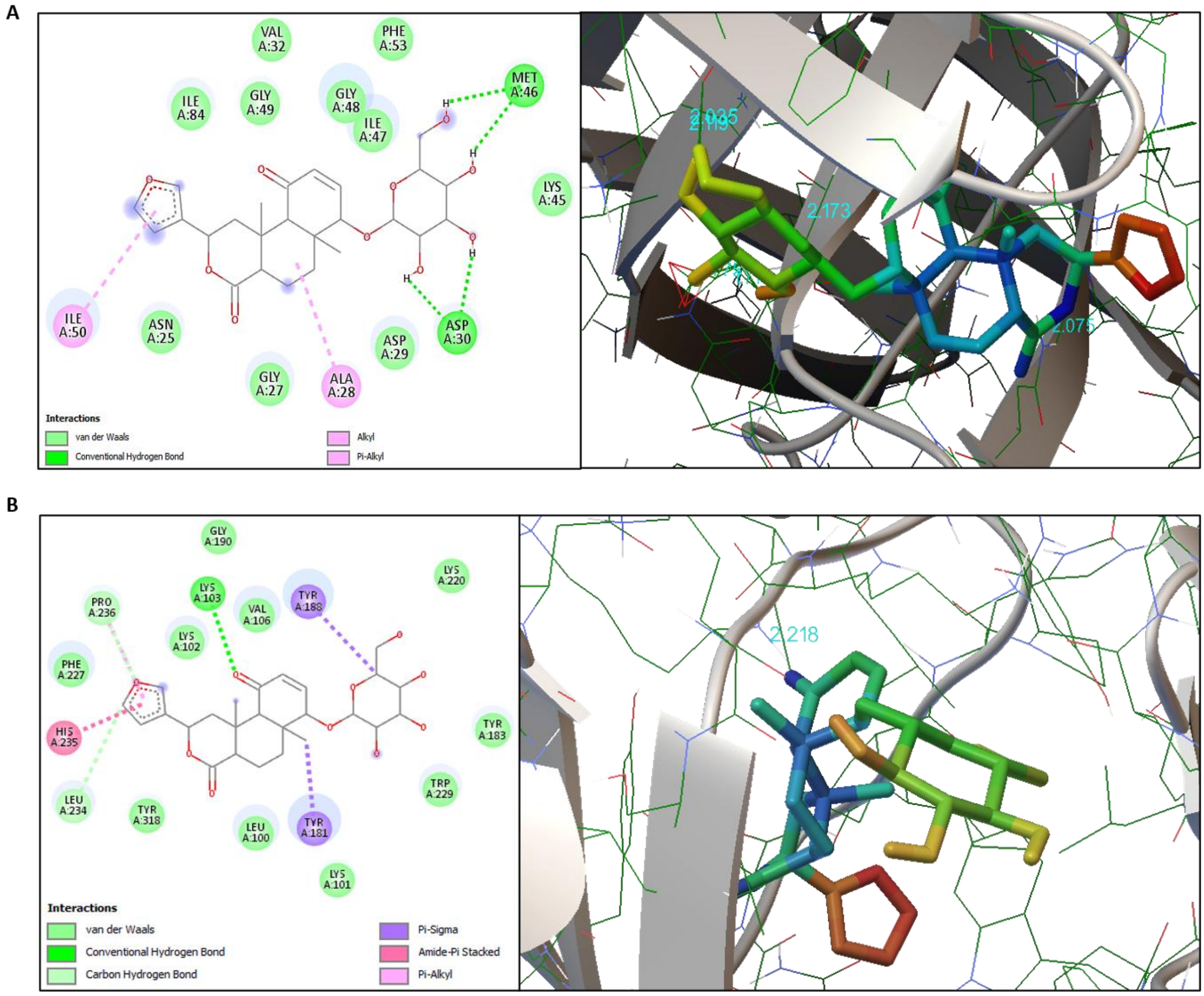

Supplementary Figure 3: Molecular interactions between phytomolecule of *Tinospora cordifolia* and tinosporaside (CID_14194109) with HIV-1 key proteins. 2D interaction plot (left panel) and 3D map (right panel) of molecular docking simulation after (A) protease (PDB 5KR0) and (B) reverse transcriptase (PDB 3QO9).

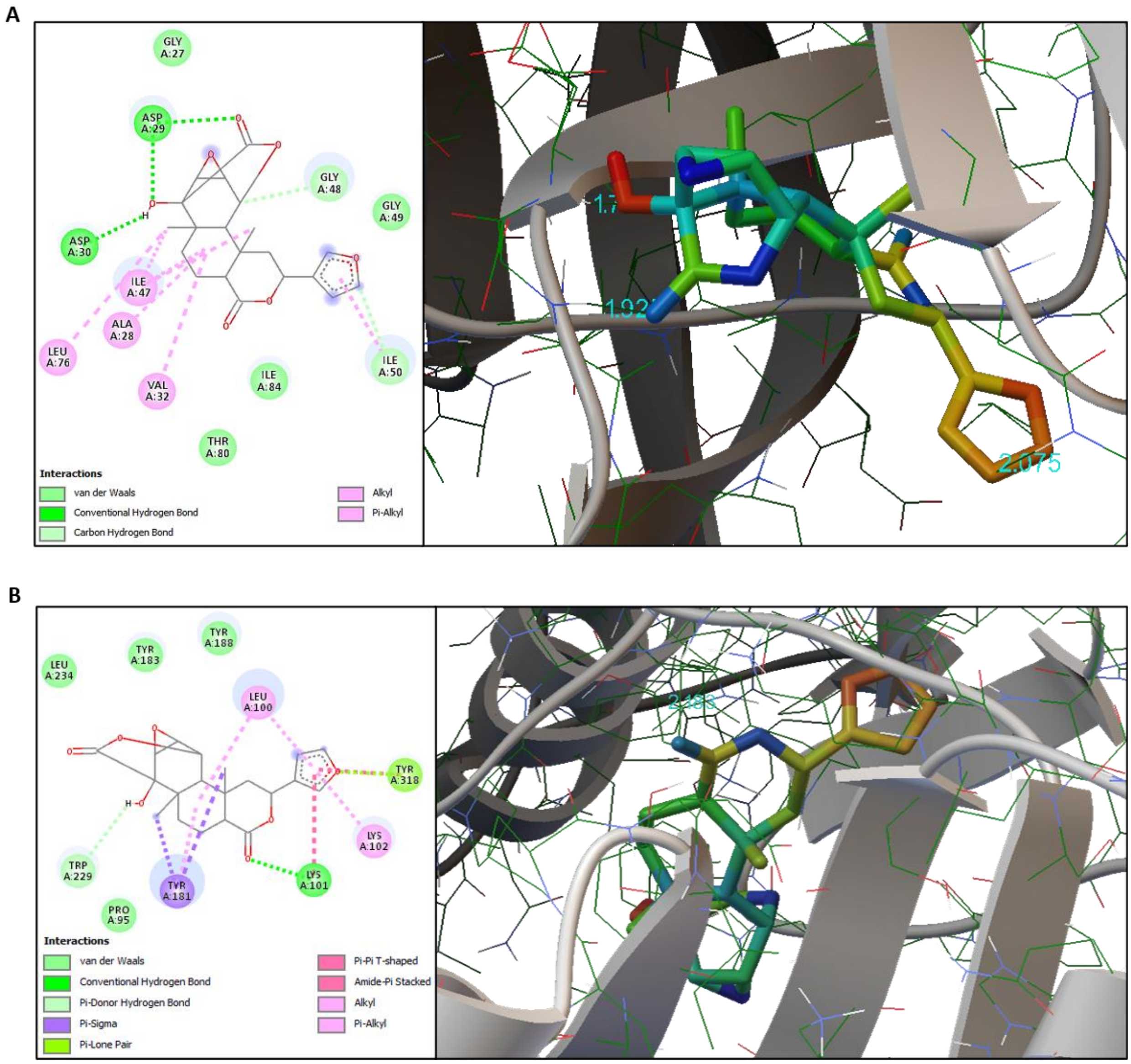

Supplementary Figure 4: Molecular interactions between phytomolecule of *Tinospora cordifolia* and tinosporide (CID_167631) with HIV-1 key proteins. 2D interaction plot (left panel) and 3D map (right panel) of molecular docking simulation after (A) protease (PDB 5KR0) and (B) reverse transcriptase (PDB 3QO9).

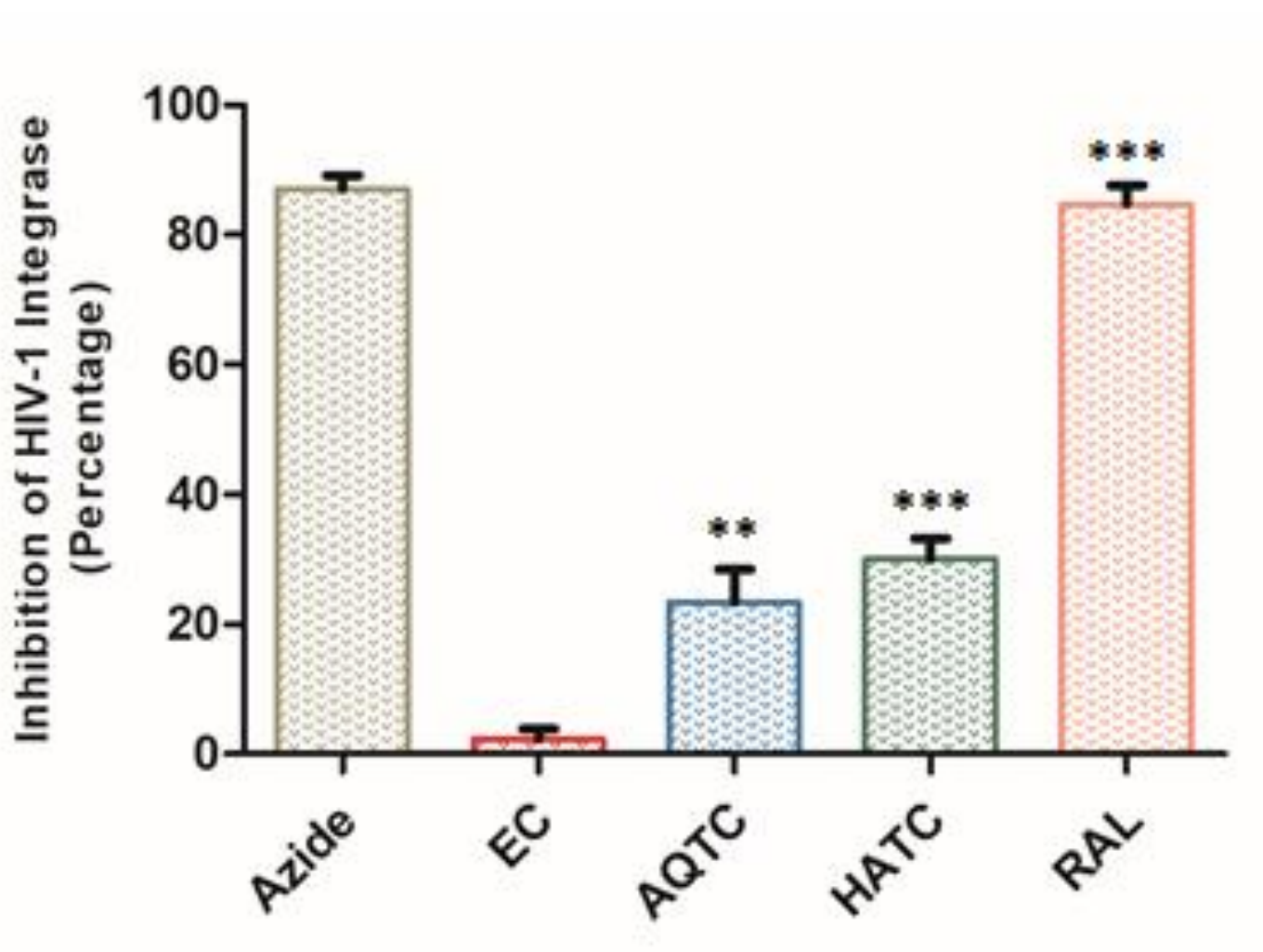

Supplementary Figure 5: The percentage of HIV-1 integrase enzyme activity suppression in the presence of *Tinospora cordifolia* aqueous extract (AQTC) and *Tinospora cordifolia* hydroalcoholic extract (HATC) was compared to the kit provided inhibitor control or azide, enzyme control or negative control (EC), and the known standard drug for HIV-1 integrase (raltegravir). The results shown are the means of at least three experimental replicates plus the standard deviations were calculated and represented as the error bar.

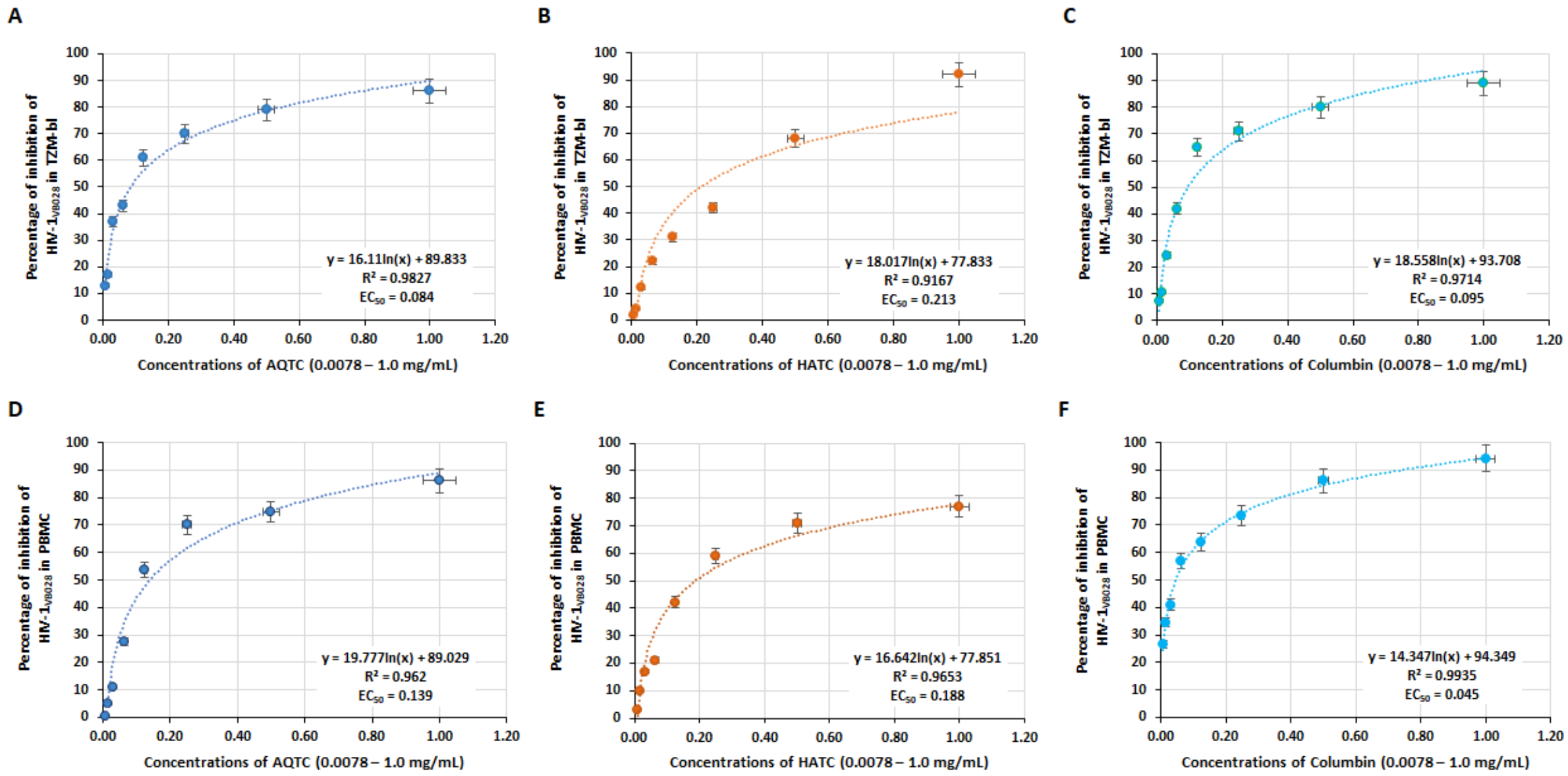

Supplementary Figure 6: Antiviral activity of *Tinospora cordifolia* extracts and columbin under high viral inoculum conditions (400 $TCID_{50}$). Dose-dependent inhibition of HIV-1_{VB028} by (A) *Tinospora cordifolia* aqueous extract (AQTC), (B) *Tinospora cordifolia* hydroalcoholic extract (HATC), and (C) columbin in TZM-bl cells. Dose-dependent inhibition of HIV-1_{VB028} by (D) AQTC, (E) HATC, and (F) columbin in primary human PBMCs (2×10^4 cells/well). Under these high infectious pressure conditions, AQTC, HATC, and columbin retained potent antiviral activity, with EC_{50} values of 0.084, 0.213, and 0.095 mg/mL in TZM-bl cells, and 0.139, 0.188, and 0.045 mg/mL in PBMCs, respectively, confirming robust antiviral efficacy against elevated viral loads in both cell line and primary human cell models.
